# Platelet membrane-camouflaged nanoparticles carry microRNA inhibitor against myocardial ischaemia‒reperfusion injury

**DOI:** 10.1186/s12951-022-01639-8

**Published:** 2022-10-04

**Authors:** Tianyi Wang, Tingting Zhou, Mingming Xu, Shuo Wang, Anqi Wu, Mingyang Zhang, You Lang Zhou, Jiahai Shi

**Affiliations:** 1 Nantong Key Laboratory of Translational Medicine in Cardiothoracic Diseases, and Research Institution of Translational Medicine in Cardiothoracic Diseases, Affiliated Hospital of Nantong University, NO.20, Xisi Road, Nantong, 226001 Jiangsu China; 2 Department of Thoracic Surgery, Affiliated Hospital of Nantong University, NO.20, Xisi Road, Nantong, 226001 Jiangsu China; 3 Department of Forensic Sciences, Soochow University, NO.178, Ganjiang Road, Suzhou, 215000 Jiangsu China; 4 Research Center of Clinical Medicine, Affiliated Hospital of Nantong University, NO.20, Xisi Road, Nantong, 226001 Jiangsu China; 5 School of Public Health, Nantong University, NO.9, Seyuan Road, Nantong, 226019 Jiangsu China

**Keywords:** Myocardial ischaemia‒reperfusion injury, Nrf2, Platelet membrane vesicles, PLGA, Delivery system

## Abstract

The incidence of myocardial ischaemia‒reperfusion injury (MIRI) is increasing every year, and there is an urgent need to develop new therapeutic approaches. Nrf2 is thought to play a protective role during MIRI and it is regulated by microRNAs (miRNAs). This study focused on PLGA nanoparticles camouflaged by platelet membrane vesicles (PMVs) (i.e., PMVs@PLGA complexes) carrying microRNA inhibitors, which regulate Nrf2 and can play a therapeutic role in the MIRI process. In vitro and in vivo characterization showed that PMVs@PLGA has excellent transfection efficiency, low toxicity and good targeting. MicroRNAs that effectively regulate Nrf2 were identified, and then PMVs@PLGA-miRNA complexes were prepared and used for in vitro and in vivo treatment. PMVs@PLGA-miRNA complexes can effectively target the delivery of inhibitors to cardiomyocytes. Our results suggest that PMVs@PLGA complexes are a novel delivery system and a novel biological approach to the treatment of MIRI.

**Supplementary Information:**

The online version contains supplementary material available at 10.1186/s12951-022-01639-8.

## Introduction

Myocardial infarction is a kind of irreversible injury caused by severe and continuous myocardial ischaemia, usually caused by thrombosis and vascular occlusion due to coronary atherosclerosis [1, 2]. The only way to rescue the ischaemic myocardium from myocardial infarction is timely reperfusion. However, in addition to saving the ischaemic myocardium from infarction, reperfusion also induces another irreversible injury, myocardial ischaemia‒reperfusion injury (MIRI) [3–5]. MIRI refers to the recovery of the blood supply after myocardial ischaemia, leading to metabolic dysfunction and structural damage. It involves complex underlying mechanisms, including excessive production of reactive oxygen species (ROS) and oxidative stress, inflammation, calcium overload, and mitochondrial dysfunction [6–9]. Increasing evidence indicates that oxidative stress is the major pathological process leading to myocardial injury after reperfusion [10].

Nuclear factor (erythroid-derived 2)-related factor 2, otherwise known as NFE2L2 or Nrf2, is indispensable in inducing endogenous antioxidant enzymes in response to oxidative stress [11]. Nrf2 is widely expressed in various oxygen-consuming organs, such as muscle, heart, blood vessels, liver, kidney and brain [12]. Existing studies have shown that Nrf2 plays a variety of important roles in the pathological process of MIRI. Nrf2 is widely involved in reducing oxidative stress, ameliorating endoplasmic reticulum stress, repairing damaged mitochondria, regulating inflammation and autophagy, etc., and it protects cardiomyocytes when MIRI occurs [13–17]. In addition, some studies have demonstrated that microRNAs (miRNAs) can regulate Nrf2 at the posttranscriptional level [18–20].

There have been many studies on applying modified miRNA inhibitors (antagomirs) directly to the body for treatment [21–23]. Intravenous injection or in situ injection of an antagomir directly antagonizes miRNA in the body, thereby enhancing the expression of a specific gene, which can play a therapeutic role. However, the short half-life and poor targeting of the antagomir itself are also difficult to ignore. The development of targeted and effective vectors is the current focus and challenge of miRNA therapy. Although viral vectors have high transfection efficiency, their cytotoxicity, carcinogenicity, and cellular immune response are poor [24, 25]. Nonviral vectors have been considered the first choice in recent years due to their high safety and biocompatibility [26, 27]. Nanoparticles have a size of approximately 100 nm, so they can easily penetrate cell membranes after being loaded with nucleic acids [28]. Moreover, this delivery system has extremely low cytotoxicity, good cell internalization ability and extremely strong transfection efficiency. Based on previous reports, this helps improve the treatment and diagnosis of cardiovascular diseases and cancers. We have previously found that biocompatible polylactic-glycolic acid (PLGA) nanoparticles have high transfection efficiency [29–31].

The incidence of MIRI has been increasing in recent years, and Nrf2 is one of the key therapeutic targets. In this study, a novel vector, a nanoparticle camouflaged by platelets that can carry inhibitors of miRNA, was prepared to indirectly increase the expression of Nrf2 by competitively binding miR-155-5p, thus acting as a myocardial protector during MIRI and providing a novel potential approach for the targeted treatment of MIRI.

## Materials and methods

### Cells and animals

H9C2 and HEK-293T cells were purchased from the National Collection of Authenticated Cell Cultures, Chinese Academy of Sciences (China); they were cultured in DMEM (high glucose) medium (01-055-1A, Biological Industries, Israel) supplemented with 10% foetal bovine serum (04-010-1A, Biological Industries, Israel). The cells were kept at 37 °C in 5% CO_2_ at atmospheric pressure.

Male Sprague Dawley rats (250 ± 10 g, 8 weeks old) were purchased from the Model Animal Research Center of Nantong University. The rats were kept in a specific pathogen-free (SPF) environment at 22 ± 1 °C, a relative humidity of 50 ± 1%, and a regular 12 h day-night cycle. All animal experiments were approved by the Medical Ethics Committee of Nantong University and conducted under the guidance of the Laboratory of Nantong University. This study was approved by the Ethics Committee of the Affiliated Hospital of Nantong University (No. S20200314-012).

### In vitro MIRI cell model and in vivo MIRI rat model

For the in vitro experiments, H9C2 cells were cultured in complete medium (DMEM with 10% FBS) to 80% confluence. The cells were then cultured in serum and sugar-free medium at 37 °C in a three-atmosphere incubator under hypoxic conditions for 6 h, which consisted of 94% N_2_, 5% CO_2_, and 1% O_2_. Finally, the cells were cultured in complete medium under regular oxygen conditions for 12 h to establish reoxidation. H9C2 cells that were not exposed to hypoxic conditions were considered the control group.

For the in vivo experiments, 8 week-old male Sprague Dawley rats were used. The rats were randomly assigned to the different groups. Animal care was conducted in accordance with the Institutional Animal Care and Use Committee (IACUC) guidelines. Preparation of the rat myocardial ischaemia reperfusion model: Rats were administered isoflurane throughout the surgery for anaesthesia. They were artificially ventilated through a tracheal intubation tube connected to a VentStar Small Animal Ventilator (R415, RWD, China). The skin of the rat's chest was depilated and disinfected with iodine. The skin was cut along the longitudinal sternum, the subcutaneous tissues and muscles were freed layer by layer with haemostatic forceps, the left 3rd to 4th intercostal space was exposed, and the dark shadow of the pulsating heart could be clearly seen. The rib cage was separated by inserting elbow haemostatic forceps into the thoracic cavity along the left edge of the sternum at the 3–4 rib space. The pericardium was cut open after opening the thoracic cavity and the heart was fully exposed with a chest opener. The left anterior descending branch of the coronary artery was ligated with 6/0 medical sutures for 30 min to block blood perfusion and then released for reperfusion, and the thoracic cavity was sutured layer by layer.

### Preparation of PMVs@PLGA-miRNA complexes

#### Preparation of platelet membrane vesicles (PMVs)

Blood from SD male rats was collected into an EDTA tube and then centrifuged at 200 × g for 20 min at room temperature (RT) to separate the red and white blood cells. The supernatant, platelet-rich plasma (PRP) containing the platelets, was collected. Phosphate-buffered saline (PBS) with 5 mM prostaglandin E1 (PGE1) (GC41905, GlpBio, USA) was added to the purified PRP to keep the platelets inactivated. The isolated platelets were then pelleted by centrifugation at 1800 ×g for 20 min at RT. After removing the supernatant, the platelets were resuspended in PBS containing 5 mM PGE1. The prepared suspended platelets were counted in a flow cytometer, and the number of platelets per tube (2.5–3.0) ×10^5^ was standardized. To fabricate PMVs, pelleted platelets from SD male rat plasma were repeatedly freeze‒thawed, centrifuged at 8000 ×g for 15 min, and then sonicated for 2 min in a bath sonicator (FS30D, Fisher Scientific, USA) [7]. The platelet membranes were filtered through a sterile 0.2 μm filter to produce the PMVs. The PMVs were aliquoted into 1 mL samples and placed at − 80 °C for storage until use. The size distribution and morphology of the PMVs were examined using transmission electron microscopy.

#### Preparation of PLGA and PLGA-miRNA complexes

PLGA nanoparticles were obtained by the double emulsion method as reported in a previous study [30, 31]. The main component of the nanoparticles is poly (D,L-lactide-co-glycolide) (PLGA, lactide:glycolide (65:35), Mw = 40–75 kDa) (P2066, Sigma‒Aldrich, USA). To load the miRNAs into the nanoparticles, the nanoparticles were modified with branched polyethyleneimine (PEI, Mw = 25 kDa) (408727, Sigma‒Aldrich, USA) to make them positively charged so they could attract the negatively charged miRNAs. The miR-140-5p, miR-144-3p, miR-155-5p, and miR-340-5p mimic, inhibitor, NC mimic, NC inhibitor, and miR-155-5p antagomir were purchased from GenePharma (China). Briefly, the nanoparticle solution was mixed with PEI in deionized water, and then the mixed solution was added to the miRNA solutions, vortexed gently and incubated for 20 min at RT to formulate the PLGA-miRNA complexes.

#### Preparation of PMVs@PLGA and PMVs@PLGA-miRNA complexes

**Fig. 1:**
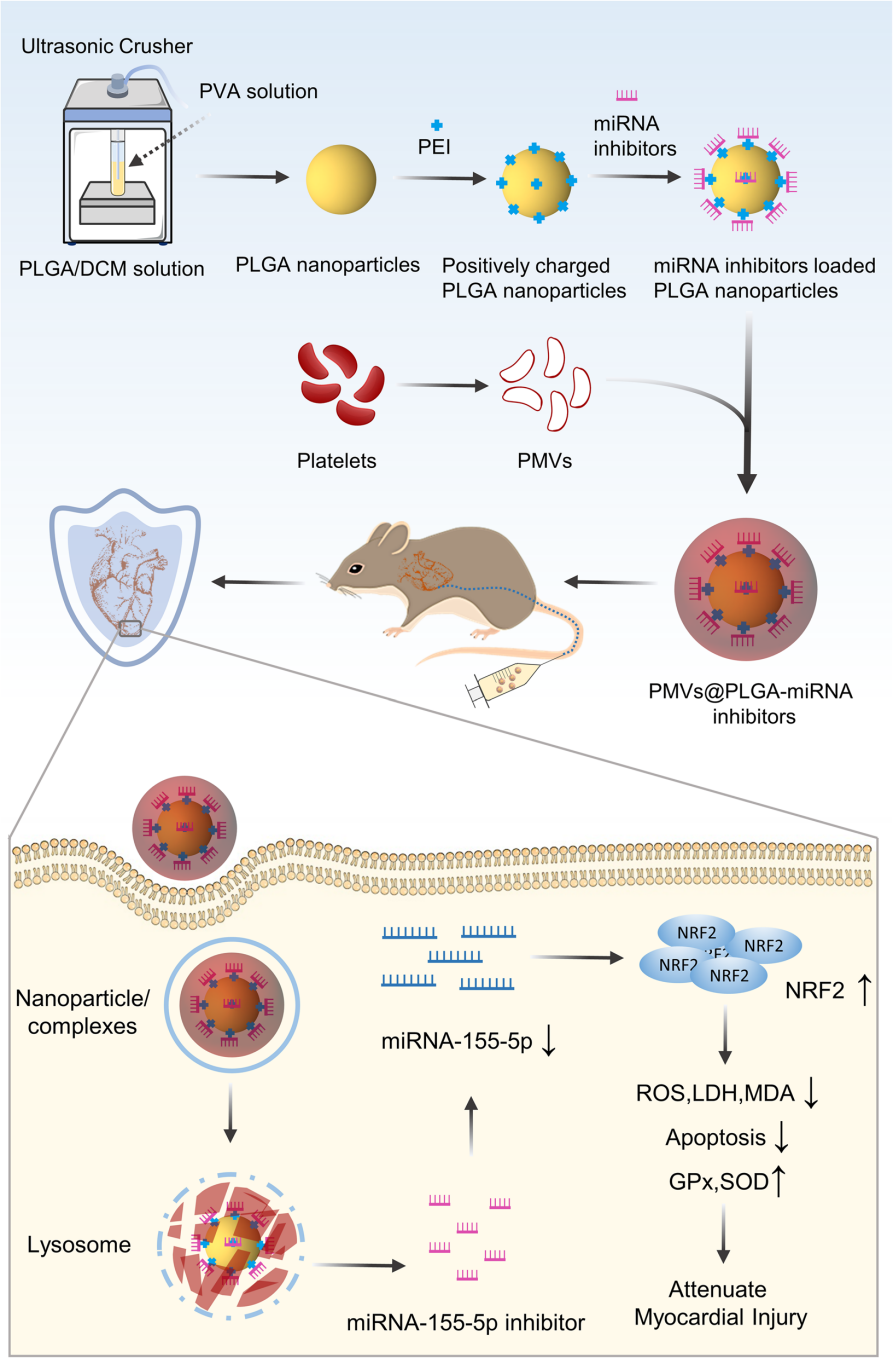
Schematic diagram of PMVs@PLGA-miRNA complexes for treating MIRI

The PMVs vesicles were fused with an equal volume of PLGA or PLGA-miRNA complexes by ultrasound (5 min, 42 kHz, 100 W). The sample was filtered 20 times using a porous syringe filter with a membrane pore size of 200 nm and centrifuged (2500 rpm, 10 min) to remove excess PMVs to produce the PMVs@PLGA or PMVs@PLGA-miRNA complexes (Fig. 1).

### Characterization of the PMVs@PLGA complexes in vitro

The characterization of the PMVs@PLGA complexes in vitro was similar to a previous study [29–31]. The size and morphology of the PLGA-miRNA complexes were characterized by transmission electron microscopy (TEM) (JEM-2100, JEOL, Japan). Briefly, the complexes were coated with platinum after freeze-drying and then imaged under TEM. The average diameter and size distribution of the complexes were determined by nanoparticle tracking analysis (NTA) (ZetaView, Particle Metrix, Germany). A gel retardation assay was used to evaluate the interaction between the PLGA nanoparticles and miRNAs at different N/M ratios (the ratio of moles of the amine groups of polyethyleneimine to the moles of the phosphate groups of RNA). The in vitro cumulative release of miRNA from the PMVs@PLGA-miRNA complexes was measured. Briefly, complexes containing 12 μg miRNA inhibitors were prepared. The complexes were equally divided into three tubes, and 50 μL PBS was added to each tube and incubated at 37 °C with gentle agitation. The supernatant was collected every two days by centrifugation at 15,000 rpm for 5 min and replaced with fresh PBS. The collected supernatant was used to detect siRNA concentrations with a Quant-iT RNA Assay Kit (Q33140, Invitrogen, USA). Finally, we calculated the release amount and plotted the miRNA release profile. A crude comparison of the proteins in platelets and the PMVs, PLGA and PMVs@PLGA complexes was performed using a Coomassie Blue Staining Kit (P0017A, Beyotime, China). The loading weight of the proteins for each lane was 20 μg. Platelet membrane markers were detected by western blotting (WB), and the main markers were CD31, CD41, CD42b, and CD61. The biocompatibility of PMVs, PLGA and PMVs@PLGA complexes with H9C2 cells was evaluated by CCK-8 assays in vitro. Thick smears were made for observation by fluorescence microscopy using PKH67-labelled PMVs and rhodamine-labelled PLGA.

When using flow cytometry to detect the particle uptake efficiency, Dil-labelled PMVs and FITC-labelled PLGA were used. The toxicity of the PMVs, PLGA and PMVs@PLGA complexes to cells was also detected by flow cytometry of H9C2 apoptosis. In addition, TUNEL apoptosis, caspase 3 activity, ROS, total SOD activity, MDA, GPx enzyme activity and LDH cytotoxicity were all used to assess their toxicity further. A haemolytic assay was also used as part of the safety check.

### Reactive oxygen species (ROS) assay

The ROS assay was completed using a Reactive Oxygen Species Assay Kit (S0033 M, Beyotime, China). Treated H9C2 cells were labelled using a DCFH-DA probe (10 μmol/L), incubated for 20 min at 37 °C, washed with PBS to remove excess probe and then assayed using flow cytometry.

### Total superoxide dismutase (SOD) activity assay

Total SOD activity was measured using a Cu/Zn-SOD and Mn-SOD Assay Kit with WST-8 (S0103, Beyotime, China). After preparing the working solution and treating the cells according to the manufacturer’s instructions, the absorbance of each sample was measured at 450 nm and the total SOD activity was calculated as described in the manual.

### Malondialdehyde (MDA) assay

MDA was measured using a Malondialdehyde (MDA) Content Test Kit (BC0020, Solarbio, China). After preparing the working solution and treating the cells according to the manufacturer’s instructions, the absorbance of each sample was measured at 532 nm and 600 nm and the MDA content was calculated as described in the manual.

### Glutathione peroxidase (GPx) enzyme activity assay

GPx enzyme activity was measured using a Total Glutathione Peroxidase Assay Kit with NADPH (S0058, Beyotime, China). After preparing the working solution and treating the cells according to the manufacturer’s instructions, the absorbance of each sample was measured at 340 nm and the GPx enzyme activity was calculated as described in the manual.

### Lactate dehydrogenase (LDH) cytotoxicity assay

LDH cytotoxicity was measured using a LDH Cytotoxicity Assay Kit (C0017, Beyotime, China). After preparing the working solution and treating the cells according to the manufacturer’s instructions, the absorbance of each sample was measured at 490 nm and 600 nm and the LDH cytotoxicity was calculated as described in the manual.

### Haemolytic assay

Blood was collected from healthy SD rats and centrifuged at 400 × g for 15 min to isolate the red blood cells. The cells were then washed 3 times with PBS, and PMVs, PLGA or PMVs@PLGA complexes suspended in PBS were mixed with the erythrocytes at a volume ratio of 6:4 and incubated for 3 h at 37 °C (a PBS-only group and a ddH_2_O group served as controls). The mixture was centrifuged, and the supernatant was transferred to a 96-well plate. The absorbance at 540 nm was measured to assess the extent of haemolysis.

### Characterization of PMVs@PLGA complexes in vivo

The metabolism of PMVs@PLGA complexes was examined using healthy SD rats to analyse the general pattern of their metabolism. For biotoxicity analysis, venous blood was collected from healthy SD rats within 1–10 days after injection of PMVs@PLGA complexes and analysed for neutrophil percentage using a fully automated haematology analyser (BC-5000 Vet, Mindray, China), liver and kidney function markers using a fully automated biochemical analyser (Vetube 30, Mindray, China), myoglobin (MYO) concentration using a Rat MYO ELISA Kit (E-EL-R0053c, Elabscience, China), a Rat Troponin I Type 3 (cTnI) ELISA Kit (E-EL-R1253c, Elabscience, China) for the cTnI concentration, and a Rat CKMB (Creatine Kinase MB Isoenzyme) ELISA Kit (E-EL-R1327c, Elabscience, China) for the CK-MB concentration. For targeting validation, rats were used to construct a MIRI model and then injected with DiR-labelled PMVs, PLGA and PMVs@PLGA complexes evaluation by live imaging. Additionally, heart tissue was collected from the rats to make frozen sections, and the toxicity of the PMVs@PLGA complexes was again verified using TUNEL apoptosis analysis and immunofluorescence (IF) analysis of BAX and BCL-2 expression.

### In vivo imaging

For the metabolic profile assay, PLGA was labelled with DiR and assembled into PMVs@PLGA complexes, suspended in PBS and injected into healthy SD rats via the tail vein. The heart, liver, spleen, lungs and kidneys were collected at 7 time points, 0, 2, 4, 6, 12, 24 and 48 h postinjection, respectively, and then rinsed appropriately with PBS, and images were taken using an animal live imaging system (ABL X5, Tanon, China) and analysed for average photons per pixel per ms (average PPP (per ms)).

For the targeting profile assay, PMVs, PLGA and PMVs@PLGA complexes were labelled with DiR and injected into SD rats via the tail vein 10 min after MIRI model construction. Twenty-four hours later, the heart, liver, spleen, lungs and kidneys of the rats were collected and washed appropriately with PBS, and images were taken using an animal live imaging system (ABL X5, Tanon, China) and analysed for average photons per pixel per ms (average PPP (per ms)).

### Flow cytometry assay

For the cell particle uptake efficiency assay, PMVs were labelled with Dil, and PLGA was labelled with FITC and assembled into PMVs@PLGA complexes. A total of 20 μg of PMVs, 20 μg of PLGA, or 20 μg of PMVs assembled with 20 μg of PLGA was added to one well of a 6-well plate in 2 mL of medium and incubated with H9C2 cells for 24 h. The H9C2 cells were collected and washed 3 times with precooled PBS, the cell concentration was adjusted to 1 × 10^6^ cells/mL, and the particle uptake efficiency was measured using a flow cytometer (FACSCalibur, BD, USA).

For the apoptosis assay, treated H9C2 cells were collected, washed three times with precooled PBS and stained with an Annexin V-Alexa Fluor 647/PI Apoptosis Assay Kit (FMSAV647, Fcmacs, China) according to the manufacturer’s instructions, followed by flow cytometry (FACSCalibur, BD, USA) to analyse the cells for apoptosis.

### TUNEL apoptosis assay

The TUNEL apoptosis assay was performed using a One-step TUNEL In Situ Apoptosis Kit (E-CK-A322, Elabscience, China). For cell samples, after 4% paraformaldehyde fixation and 0.3% Triton-X100 permeabilization, the TUNEL staining working solution was configured for staining according to the manufacturer’s instructions, and the nuclei were stained using DAPI staining solution, washed appropriately with PBS and observed with a fluorescence microscope. For frozen sections of tissue, after 4% paraformaldehyde fixation and proteinase-K permeabilization, the TUNEL staining working solution was configured for staining according to the manufacturer’s instructions, and the nuclei were stained using DAPI staining solution, washed appropriately with PBS and observed with a fluorescence microscope.

### Caspase-3 activity assay

The caspase-3 activity assay was performed using a GreenNuc Caspase-3 Assay Kit for Live Cells (C1168 M, Beyotime, China). After staining treated live cells with GreenNuc staining solution configured according to the manufacturer’s instructions, they were fixed using 4% paraformaldehyde and permeabilized with 0.3% Triton X-100. The nuclei were subsequently stained using Hoechst staining solution and observed using fluorescence microscopy.

### qPCR

Total RNA was isolated from cells using Invitrogen TRIzol reagent (15596026, Thermo Fisher Scientific, USA). For mRNA analysis, a reverse transcription reaction was performed via the RevertAid First Strand cDNA Synthesis Kit (K1622, Thermo Fisher Scientific) according to the manufacturer’s instructions. qPCR was performed using PowerUp SYBR Green Master Mix (A25742, Thermo Fisher Scientific, USA) on a QuantStudio 5 Real-Time PCR System (A28569, Thermo Fisher Scientific, USA). All target genes were normalized to GAPDH. The primer sequences used are listed as follows:

rno-Nrf2 forward, 5′-TCCAAGTCCAGAAGCCAAACTGAC-3′;

rno-Nrf2 reverse, 5′-GGAGAGGATGCTGAAGGAATC-3′;

rno-GAPDH forward, 5′-GACATGCCGCCTGGAGAAAC-3′;

rno-GAPDH reverse, 5′-AGCCCAGGATGCCCTTTAGT-3′.

For miRNA analysis, a reverse transcription reaction was performed via an EZ-press microRNA Reverse Transcription Kit (miRT2-L, EZBioscience, USA) according to the manufacturer’s instructions. qPCR was performed using an EZ-press microRNA qPCR Kit (ROX2 plus) (miQP2, EZBioscience, USA) on a QuantStudio 5 Real-Time PCR System. All target genes were standardized to U6. The primer sequences used are listed as follows:

rno-miR-144-3p forward, 5′-GCGCGCGTACAGTATAGATGA-3′;

rno-miR-155-5p forward, 5′-GCGCGTTAATGCTAATTGTGAT-3′;

rno-miR-140-5p forward, 5′-CGCGCAGTGGTTTTACCCTA-3′;

rno-miR-153-3p forward, 5′-CGCGTTGCATAGTCACAAAA-3′;

rno-miR-410-3p forward, 5′-CGCGGCAATTTAGTGTGTGT-3′;

rno-miR-27a-3p forward, 5′-TTCACAGTGGCTAAGTTCCGC-3′;

rno-miR-27b-3p forward, 5′-TTCACAGTGGCTAAGTTCTGC-3′;

rno-miR-340-5p forward, 5′-GCGCGTTATAAAGCAATGAGA-3′;

rno-miR-106b-5p forward, 5′-TAAAGTGCTGACAGTGCAGAT-3′;

rno-miR-495 forward, 5′-AAACAAACATGGTGCACTTCTT-3′;

rno-miR-142-5p forward, 5′-GCGCGCATAAAGTAGAAAGC-3′;

rno-miR-17-5p forward, 5′-CAAAGTGCTTACAGTGCAGGTAG-3′;

rno-miR-93-5p forward, 5′- CAAAGTGCTGTTCGTGCAGGTAG-3′;

rno-miR-128-3p forward, 5′-CGCGTCACAGTGAACCGGT-3′;

U6 forward, 5’-CCTGCTTCGGCAGCACA-3’.

The reverse primers for the miRNAs and U6 were provided in the EZ-press microRNA qPCR Kit (ROX2 plus). Quantification of qPCR results was performed by the 2^−ΔΔCt^ method.

### Transfection of miRNA mimic, inhibitor and antagomir

For in vitro transfection, one well of a 6-well plate was used. After the cells grew to 60–80% confluence, 20 μg of PMVs, 20 μg of PLGA and 200 pmol of miRNA in 125 μL of basal medium were assembled into PMVs@PLGA-miRNA complexes and mixed thoroughly and gently. The cells were allowed to stand for 15 min and then added to the complete medium in the 6-well plate to complete the transfection.

For in vivo transfection, each rat was prepared with antagomir at 10 mg/kg for transfection. The corresponding weight of antagomir was made into PMVs@PLGA-miRNA antagomir in vitro. Approximately 10 min after the completion of the MIRI model, it was injected into the rat via the tail vein to complete the transfection.

### Dual-luciferase reporter gene assays

HEK-293T cells were seeded into a 24-well plate, incubated overnight, and then transfected with the dual luciferase plasmid vectors and miR-140-5p, miR-144-3p, miR-155-5p, and miR-340-5p mimics. According to the manufacturer's protocol, the luciferase activity was checked by a FLUOROSKAN FL SYSTEM (5200220, Thermo Fisher Scientific, USA) using the Dual-Luciferase Reporter Assay System (E1910, Promega, USA). The firefly luciferase activity was normalized to the Renilla luciferase activity in each well.

### Western blot (WB) analysis and immunofluorescence (IF) analysis

Cell pellets or tissue homogenates were lysed with RIPA lysis buffer (WB3100, NCM Biotech, China) and PMSF (P0100, Solarbio, China) for 1 h; after centrifugation at 12,000 rpm for 15 min, the supernatant was aspirated, mixed with loading buffer, and incubated at 100 °C for 10 min to obtain protein samples. Western blot (WB) experiments were performed using SDS‒PAGE. The primary antibodies used were anti-Nrf2 (1:1000) (T55136, Abmart, China), anti-β-actin (1:10,000) (AC026, ABclonal, China), CD31 (1:1000) (11,265–1-AP, Proteintech, China), anti-CD41 (1:1000) (18,308–1-AP, Proteintech, China), anti-CD42b (1:1000) (12,860–1-AP, Proteintech, China), and anti-CD61 (1:1000) (18,309–1-AP, Proteintech, China). The secondary antibody was HRP-conjugated goat anti-rabbit IgG (1:10,000) (RS0002, Immunoway, USA). Proteins were detected by a chemiluminescent imaging system (ChemDoc MP, BIO-RAD, USA), and greyscale analysis was performed using ImageJ software.

Frozen sections were used for immunofluorescence (IF). After 0.3% Triton X-100 permeabilization and 5% BSA blocking, the slides were incubated overnight with anti-Nrf2 (1:100) (T55136, Abmart, China), anti-BAX (1:100) (50,599-2-Ig, Proteintech, China), and anti-BCL-2 (1:100) (26,593-1-AP, Proteintech, China) antibodies at 4 °C. The sections were incubated with the secondary antibody CoraLite594-conjugated goat anti-rabbit IgG (1:500) (SA00013-4, Proteintech, China) at RT for 1 h, and the nuclei were stained with DAPI (C1002, Beyotime, China).

### Triphenyl tetrazolium chloride (TTC) staining

After model preparation was completed or treatment was completed, the heart was removed, rinsed in saline, sliced into 2 mm sections and placed in 2% TTC solution (G3005, Solarbio, China) and incubated at 37 °C in the dark for 30 min. The heart sections were then fixed in 4% paraformaldehyde for 6 h, photographed and then analysed for the size of the white areas.

### Statistical analysis

The data were evaluated by GraphPad Prism software and expressed as the mean ± SD. Differences between groups were assessed by one-way analysis of variance and subsequent Tukey posttests. P < 0.05 indicates significance.

## Results

### Characterization of PMVs@PLGA complexes in vitro

**Fig. 2:**
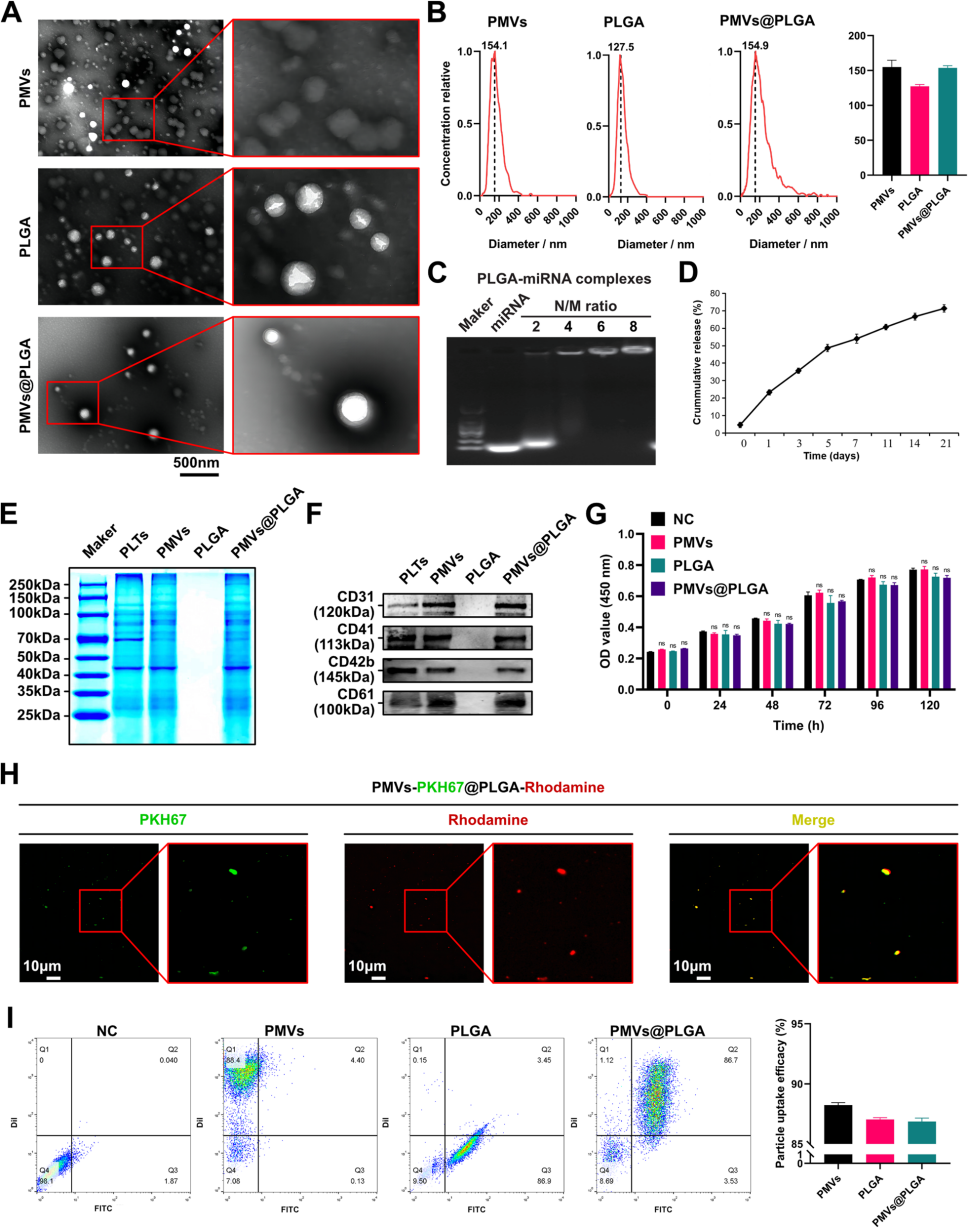
Characterization of PMVs@PLGA complexes in vitro. **A** Transmission electron microscopy images of PMVs, PLGA and PMVs@PLGA complexes (scale bars indicate 500 nm). **B** NTA analysis of PMVs, PLGA and PMVs@PLGA complexes. **C** Agarose gel electrophoresis to identify complexes with different N/M ratios (N: nanoparticles; M: miRNAs). **D** In vitro cumulative release profiles of the miRNA from the PLGA-miRNA complexes in PBS at pH 7.2. **E** Protein Coomassie blue staining images of PMVs, PLGA and PMVs@PLGA complexes. **F** Detection of key platelet membrane markers on PMVs, PLGA and PMVs@PLGA complexes. **G** Validation of PMVs, PLGA and PMVs@PLGA complexes against H9C2 cytotoxicity by CCK-8 assays. **H** Fluorescent micrographs of thick smears of PMVs@PLGA (PMVs labelled using PKH67, PLGA labelled using rhodamine) (scale bars indicate 10 μm). **I** Flow cytometry assay for particle uptake efficiency of PMVs, PLGA and PMVs@PLGA complexes into H9C2 cells (PMVs using Dil labelling, PLGA using FITC labelling). ns no significance

After preparing PMVs and PLGA and assembling them together as PMVs@PLGA complexes, we observed them using TEM. The TEM observations showed that the PMVs we prepared were vesicle-like with a relatively uniform particle size, and the PLGA were spherical vesicles of a relatively uniform particle size, while the assembled PMVs@PLGA complexes clearly showed a distinct bilayer structure. The PMVs encapsulated the PLGA to form a homogeneous spherical vesicle complex of uniform particle size **(**Fig. 2A**)**. We then analysed their particle size using the NTA system, and the results showed that both PMVs, PLGA, and the assembled PMVs@PLGA complexes had homogeneous particle sizes. The particle size of PMVs is approximately 155 ± 15 nm, PLGA is approximately 125 ± 5 nm and PMVs@PLGA is approximately 155 ± 5 nm **(**Fig. 2B**)**.

We then matched the nanoparticles and miRNA inhibitor/mimic/antagomir in different mass ratios and performed gel retardation experiments. The results showed that the best N/M ratio of nanoparticles to miRNA inhibitor/mimic/antagomir was 4:1. Nanoparticles containing miRNA showed excellent retardation at this ratio (Fig. 2C). The zeta potential of the complex (N/M ratio is 4:1) is approximately 32.6 millivolts.

The slow-release curve of the miRNA from the PLGA-miRNA complexes was detected, and approximately 80% of the loaded miRNA was released within 21 days (Fig. 2D).

To verify the assembly process of PMVs@PLGA complexes could retain the key proteins of PMVs themselves, we extracted the total proteins of PLTs, PMVs, PLGA and PMVs@PLGA complexes, and by Coomassie Blue staining experiments, we found that the protein bands of PLTs and PMVs were significantly different, while the protein bands of PMVs and PMVs@PLGA complexes were not significantly different (Fig. 2E). Furthermore, we examined four typical markers on platelet membranes (CD31, CD41, CD42b, CD61) using WB and found that PMVs and PMVs@PLGA complexes had good retention of these markers, suggesting that PMVs@PLGA complexes may retain the biological functions of PLTs themselves (Fig. 2F).

We then tested their cytotoxicity using CCK-8 assays and found that PMVs, PLGA and PMVs@PLGA complexes were not significantly toxic to H9C2 cells over a 120 h period (Fig. 2G).

We used PKH67 to label PMVs and rhodamine to label PLGA, assembled PMVs@PLGA complexes and then smeared and observed them using microscopy. The results showed that the fluorescent moieties could label PMVs@PLGA complexes very well and side by side, and we had an effective assembly of PMVs and PLGA (Fig. 2H). Subsequently, we also transfected H9C2 cells using Dil-labelled PMVs and FITC-labelled PLGA and assembled them into PMVs@PLGA complexes and tested the particle uptake efficiency using flow cytometry (using an equal volume of PBS as NC). The results showed that both PMVs and PLGA had good particle uptake efficiency and that assembling them into PMVs@PLGA complexes did not affect their particle uptake efficiency; the particle uptake efficiency of all three was close to 90% (Fig. 2I).

**Fig. 3:**
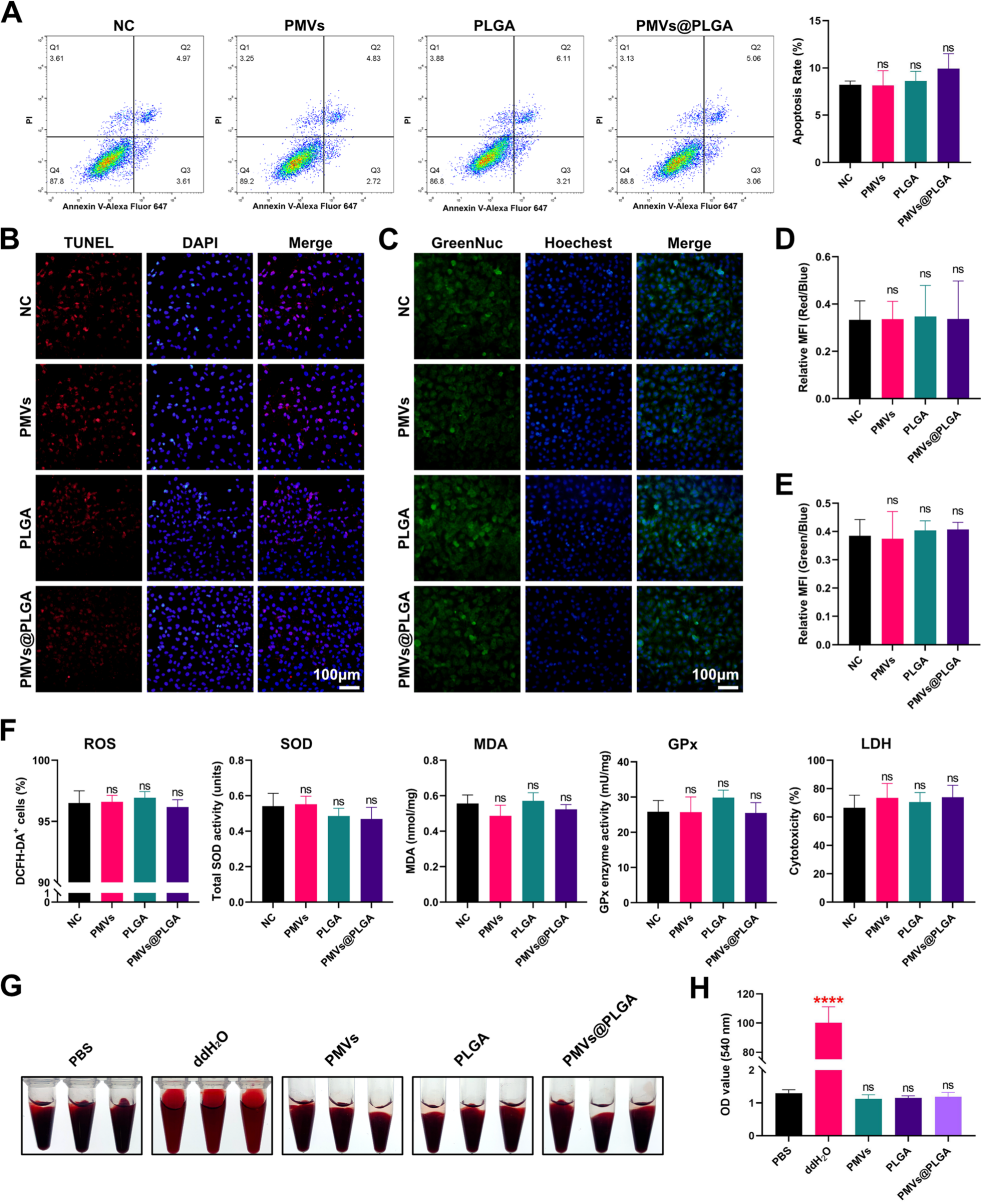
Toxicity assessment of PMVs@PLGA complexes in vitro. **A** Flow cytometry detection of the effect of PMVs, PLGA and PMVs@PLGA on the apoptosis of H9C2 cells. **B** Fluorescent micrographs of TUNEL apoptosis of H9C2 cells after exposure to PMVs, PLGA or PMVs@PLGA (scale bars indicate 100 μm). **C** Fluorescence micrographs of the effects of PMVs, PLGA and PMVs@PLGA on caspase 3 activity in H9C2 cells (scale bars indicate 100 μm). **D** Results of the statistical analysis of the effects of PMVs, PLGA and PMVs@PLGA on TUNEL apoptosis of H9C2 cells. **E** Results of the statistical analysis of the caspase 3 activity in H9C2 cells after exposure to PMVs, PLGA or PMVs@PLGA. (F) Effect of PMVs, PLGA and PMVs@PLGA on ROS, total SOD activity, MDA, GPx enzyme activity and LDH cytotoxicity in H9C2 cells. **G** Photographs of the haemolytic assay of PMVs, PLGA and PMVs@PLGA on erythrocytes from healthy SD rats. **H** PMVs, PLGA and PMVs@PLGA in the haemolysis assay supernatant of healthy SD rat erythrocytes at 540 nm absorbance. *ns* no significance; ****P < 0.0001

To examine the toxicity of PMVs@PLGA complexes in vitro, we treated H9C2 cells with PMVs, PLGA and PMVs@PLGA complexes and observed their apoptosis rates by flow cytometry (using an equal volume of PBS as NC). The results showed that the PMVs, PLGA and PMVs@PLGA complexes had no significant effect on the apoptosis of H9C2 cells (Fig. 3A). We also examined TUNEL apoptosis and caspase 3 activity in H9C2 cells (using an equal volume of PBS as NC). Fluorescence microscopy results showed that PMVs, PLGA and PMVs@PLGA complexes had no significant effect on TUNEL apoptosis or caspase 3 activity in H9C2 cells (Fig. 3B–C). The same conclusion was reached after analysis of the relative fluorescence intensity using ImageJ (Fig. 3D–E).

Subsequently, we examined ROS, total SOD activity, MDA, GPx enzyme activity and LDH cytotoxicity in transfected H9C2 cells (using an equal volume of PBS as NC), and all results showed that PMVs, PLGA and PMVs@PLGA complexes were safe and nontoxic to H9C2 cells (Fig. 3F).

Finally, to test whether PMVs, PLGA and PMVs@PLGA complexes cause haemolysis, we coincubated them with rat erythrocytes. We observed that after coincubation with PMVs, PLGA and PMVs@PLGA complexes, the supernatant remained clear and showed no signs of haemolysis (Fig. 3G). The 540 nm absorbance test of the supernatant also showed that PMVs, PLGA and PMVs@PLGA complexes did not cause haemolysis of rat erythrocytes (Fig. 3H).

The above results show that PMVs@PLGA complexes have homogeneous particle sizes, good transfection efficiency while retaining important proteins from the platelet membranes and are safe and nontoxic to cells, making them ideal transfection vectors in vivo and in vitro.

### Characterization of PMVs@PLGA complexes in vivo

**Fig. 4:**
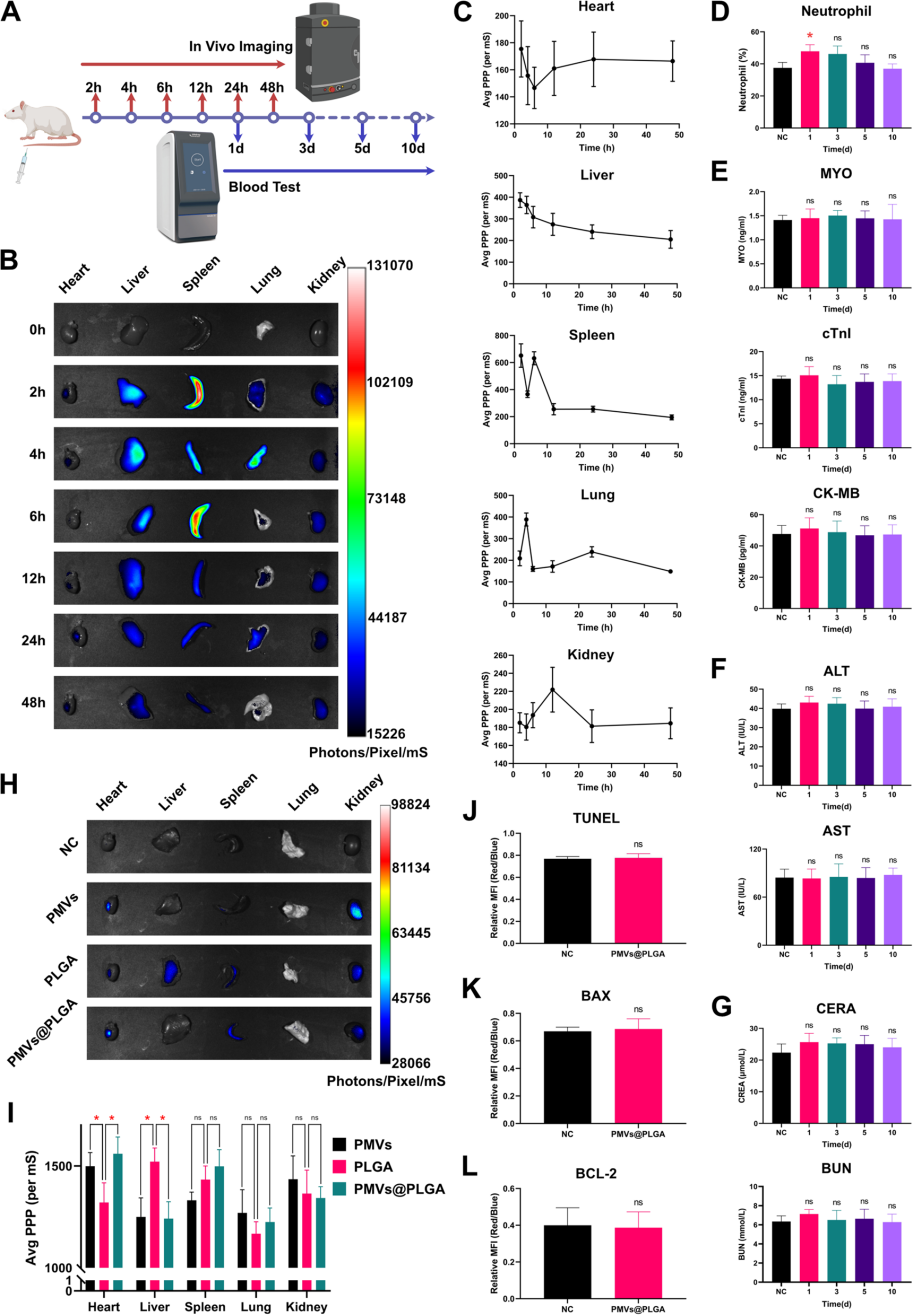
Characterization of PMVs@PLGA complexes in vivo. **A** Flow chart of the in vivo metabolism of the PMVs@PLGA complex and its toxicity assessment. **B** In vivo images of the metabolism of the PMVs@PLGA complex in healthy SD rats. **C** Quantitative analysis of the metabolism of the PMVs@PLGA complex in the heart, liver, spleen, lungs and kidneys of healthy SD rats. **D** Effect of the PMVs@PLGA complex on the percentage of neutrophils in healthy SD rats. **E** Effect of the PMVs@PLGA complex on cardiac function in healthy SD rats. **F** Effect of the PMVs@PLGA complex on liver function in healthy SD rats. **G** Effect of the PMVs@PLGA complex on renal function in healthy SD rats. **H** In vivo images of PMVs, PLGA and PMVs@PLGA complexes in the MIRI model constructed after in vivo validation of targeting in SD rats. **I** Quantitative analysis of PMVs, PLGAs and PMVs@PLGA complexes in the MIRI model constructed after in vivo validation of targeting in SD rats. (**J**) Quantitative analysis of the TUNEL apoptosis assay in cardiomyocytes after targeting the PMVs@PLGA complexes to the heart of SD MIRI model rats. **K** Quantitative analysis of the BAX IF assay of cardiomyocytes after targeting the PMVs@PLGA complexes to the heart of SD MIRI model rats. **L** Quantitative analysis of BCL-2 IF in cardiomyocytes after targeting the PMVs@PLGA complexes to the heart of SD MIRI model rats. *ns* no significance; *P < 0.05

Having fully demonstrated the safety of PMVs, PLGAs and PMVs@PLGA complexes in vitro, we started to characterize them in vivo to validate their safety and targeting ability (Fig. 4A). We labelled PLGA with DiR and assembled it with PMVs to form PMVs@PLGA complexes, which were injected into healthy rats via the tail vein and imaged in vivo at different time points. The results showed that PMVs@PLGA complexes were significantly enriched in the liver, spleen and kidney of the rats 2–48 h after injection, probably due to the biological properties of the PMVs themselves (Fig. 4B). By analysing the average photons per pixel per ms (average PPP (per ms)) of the five organs, we found that the liver and kidney, as the main metabolic organs, play a key role in the metabolism of PMVs@PLGA complexes. The liver had the highest average PPP (per ms) at 2 h postinjection, followed by a gradual decrease, while the kidney had a peak at 12 h postinjection, followed by a gradual decrease. There was no significant pattern in the average PPP (per ms) in other organs, as they are not metabolizing organs (Fig. 4C).

To test whether PMVs@PLGA complexes cause damage to the heart, liver and kidneys, venous blood was collected from rats at different time points, and a number of recognized biomarkers were tested (using an equal volume of PBS as NC). The results showed that neutrophils were abnormally elevated 1 day after injection and then quickly returned to normal, which may be a normal response of the rat's immune system to the PMVs@PLGA complexes injected into the body (Fig. 4D). However, other biomarkers (MYO, cTnI, CK-MB for cardiotoxicity; ALT, AST for hepatotoxicity; CERA, BUN for nephrotoxicity) did not change significantly between 1–10 days after injection (Fig. 4E–G). This indicates that PMVs@PLGA complexes are safe and nontoxic to rats.

In previous studies, we verified that PMVs are well targeted to the site of injury in the heart as carriers in the treatment of MIRI [32]. Here, we used DiR to label PMVs and PLGA and assembled them into PMVs@PLGA complexes, validating their targeting in MIRI rats (using an equal volume of PBS as NC). We performed in vivo imaging 24 h after injection, and the results showed that PMVs had good targeting of the heart, whereas PLGA did not. After assembly, the PMVs@PLGA complexes retained the targeting properties of the PMVs very well (Fig. 4H). By analysing the average PPP (per ms) from five organs, we found that PMVs and PMVs@PLGA complexes could be enriched in the heart 24 h after injection, while untargeted PLGA quickly entered the liver for metabolism (Fig. 4I).

To determine whether PMVs@PLGA complexes are toxic to the heart, we performed TUNEL apoptosis assays as well as IF assays for BAX and BCL-2 at sites of myocardial injury where targeting was previously validated. The results showed that the PMVs@PLGA complexes had no significant effect on TUNEL apoptosis or the expression of BAX and BCL-2 at the sites of cardiac injury (Fig. 4J–L, Additional file 1: Fig. S1A–C).

All of these results indicate that PMVs@PLGA complexes are safe and nontoxic in rats and have good targeting properties. Considering this along with their good transfection efficiency previously demonstrated in vitro, PMVs@PLGA complexes are a potential therapeutic vector for MIRI.

### MiRNA regulation of Nrf2 in MIRI

**Fig. 5:**
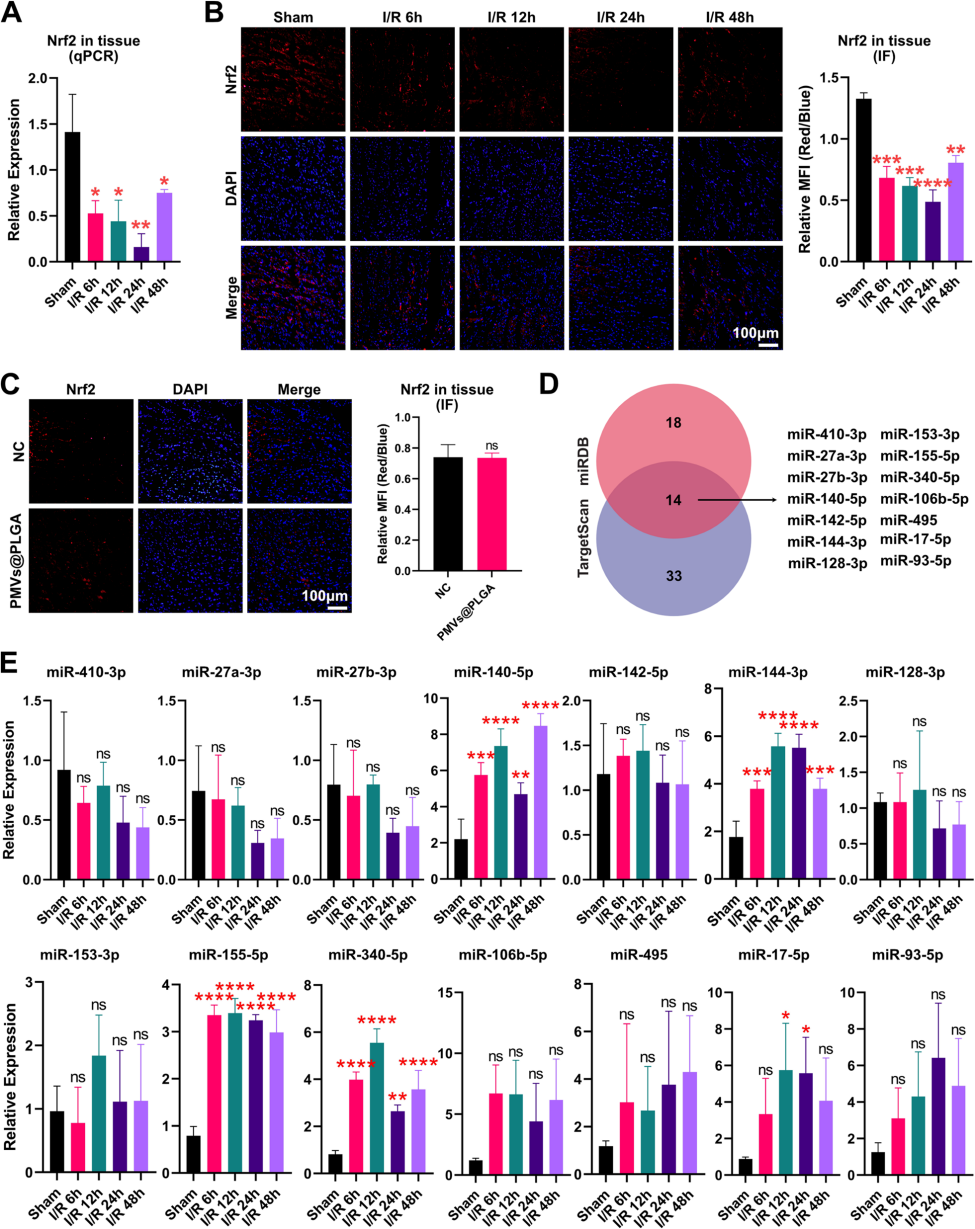
MiRNA regulation of Nrf2 in MIRI (1). **A** qPCR assay of Nrf2 expression in tissues. **B** Fluorescence micrographs and quantitative analysis of IF detection of Nrf2 expression in tissues (scale bars indicate 100 μm). **C** Fluorescence micrographs and quantitative analysis of IF assays for Nrf2 expression in PMVs@PLGA-treated myocardium (scale bars indicate 100 μm). **D** Prediction results for Nrf2 mRNA-binding miRNA from two databases, TargetScan and miRDB. **E** qPCR assay of 14 miRNAs expressed in tissues. *ns* no significance; *P < 0.05; **P < 0.01; *** P < 0.001; **** P < 0.0001

Many studies have shown that Nrf2 plays a protective role in MIRI [33]. We have also confirmed in previous studies that the activation of Nrf2 can reduce oxidative stress during MIRI by inducing the secretion of anti-inflammatory factors [34]. Here, we again used qPCR to confirm that Nrf2 expression in myocardial tissue was significantly reduced at different time points after the onset of MIRI (Fig. 5A). We also detected the expression of Nrf2 in myocardial tissues using IF and came to the same conclusion (Fig. 5B).

Meanwhile, we detected the expression of Nrf2 in myocardial tissues by IF 24 h after injection of PMVs@PLGA complexes in rats with MIRI, and we found that the PMVs@PLGA complexes did not affect the expression of Nrf2 (Fig. 5C). Therefore, how to increase the expression of Nrf2 after the onset of MIRI is the focus of the rest of this study.

We searched two databases, miRDB and TargetScan, and identified 32 and 47 potential miRNAs, respectively, that could bind to Nrf2 mRNA. Of these, a total of 14 miRNAs appeared at the intersection of the predicted results from the two databases (Fig. 5D). Subsequently, we used qPCR to detect changes in the expression levels of these 14 miRNAs in rat heart tissue after MIRI and found that the expression levels of only four miRNAs were elevated compared to the sham-operated group, namely, miR-144-3p, miR-155-5p, miR-140-5p, and miR-340-5p (Fig. 5E).

**Fig. 6:**
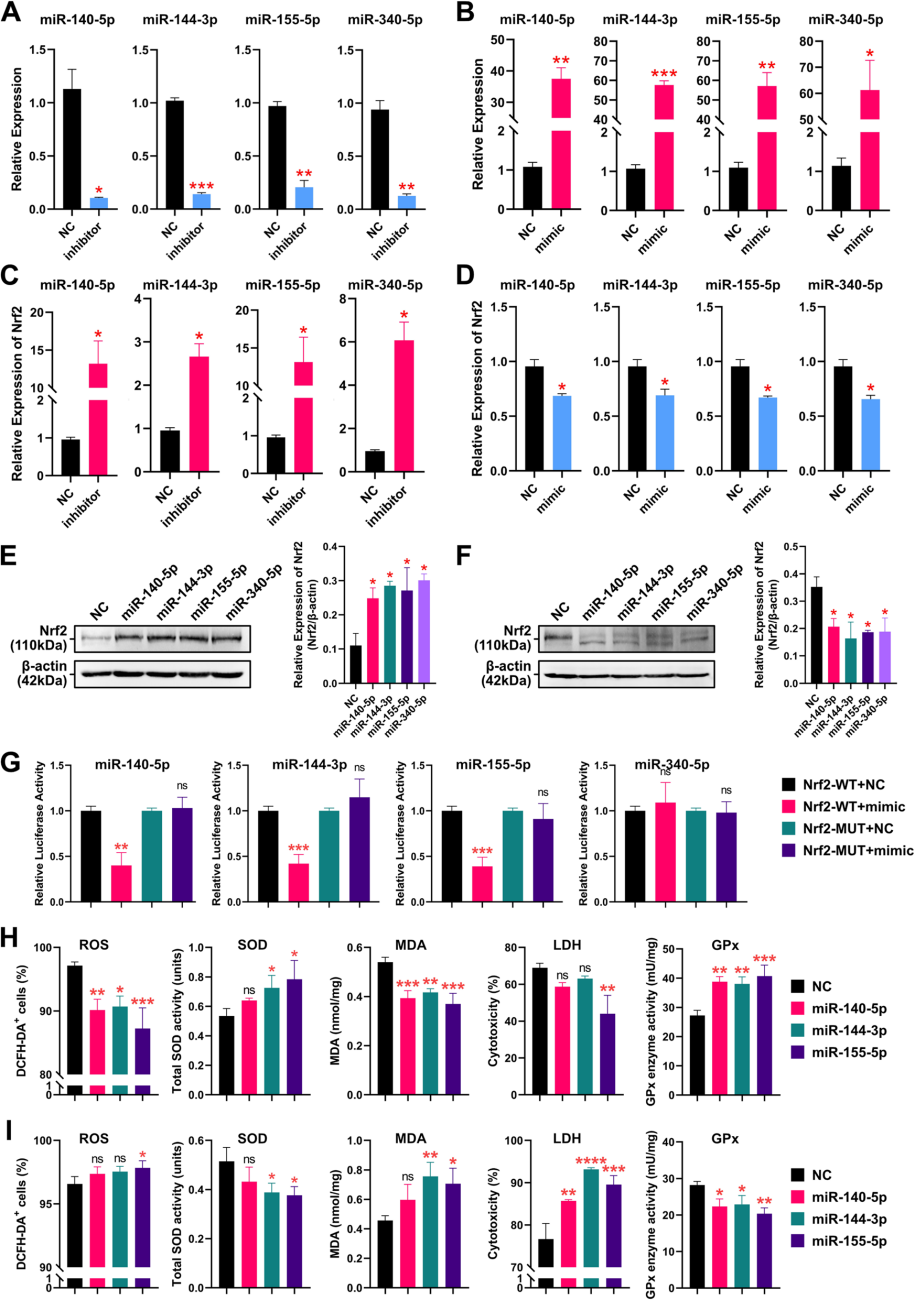
MiRNA regulation of Nrf2 in MIRI (2). **A** Interference efficiency of the inhibitor of 4 miRNAs. **B** Overexpression efficiency of mimics of the 4 miRNAs. **C** qPCR detection of Nrf2 expression after interference with the 4 miRNAs. **D** qPCR detection of Nrf2 expression after overexpression of the 4 miRNAs. **E** WB detection of Nrf2 expression after interference with the 4 miRNAs. **F** WB detection of Nrf2 expression after overexpression of the 4 miRNAs. **G** Dual luciferase reporter gene assay of the 4 miRNAs with Nrf2. **H** Assessment of ROS, total SOD activity, MDA, GPx enzyme activity and LDH cytotoxicity in H9C2 cells after interference with the 3 miRNAs. **I** Assessment of ROS, total SOD activity, MDA, GPx enzyme activity and LDH cytotoxicity in H9C2 cells after overexpression of the 3 miRNAs. *ns* no significance; *P < 0.05; **P < 0.01; *** P < 0.001; **** P < 0.0001

To further explore the regulatory relationship between these four miRNAs and Nrf2, we interfered with and overexpressed these four miRNAs using inhibitors and mimics (PMVs@PLGA complexes were used as the transfection vectors) and examined the changes in interference/overexpression efficiency and Nrf2 expression levels using qPCR (using inhibitor NC or mimic NC as NC). The results showed that all four miRNA inhibitors achieved significant interference, while the mimics all played a significant role in their overexpression (Fig. 6A–B). Meanwhile, the expression of Nrf2 significantly increased after interfering with these 4 miRNAs and decreased significantly after overexpressing these 4 miRNAs (using inhibitor NC or mimic NC as NC) (Fig. 6C–D). WBs confirmed these results (using inhibitor NC or mimic NC as NC) (Fig. 6E–F). The above results suggest that four miRNAs, miR-144-3p, miR-155-5p, miR-140-5p and miR-340-5p, may bind to Nrf2 mRNA after the onset of MIRI and reduce the expression of Nrf2, thus playing an injurious role in the process of MIRI.

Subsequently, we constructed a dual luciferase vector and performed the assay using HEK-293T cells (using mimic NC as NC) (Additional file 2: Fig. S2). The results showed that overexpression of miR-140-5p, miR-144-3p and miR-155-5p resulted in a significant decrease in luciferase activity, whereas overexpression of miR-340-5p resulted in no significant change in luciferase activity. This finding suggests that only miR-140-5p, miR-144-3p and miR-155-5p directly bind to Nrf2 mRNA (Fig. 6G). Furthermore, we examined ROS, total SOD activity, MDA, GPx enzyme activity and LDH cytotoxicity in H9C2 cells after disrupting and overexpressing miR-140-5p, miR-144-3p and miR-155-5p. The results showed that the simulated MIRI using H9C2 cells was reduced by disrupting these three miRNAs, as evidenced by the decrease in ROS, increase in total SOD activity, decrease in MDA, increase in GPx enzyme activity and decrease in LDH cytotoxicity (Fig. 6H). The opposite effect was observed after overexpression of these three miRNAs (Fig. 6I). Notably, interference with miR-155-5p had the most significant therapeutic effect on H9C2 cells.

### MiR-155-5p plays a key role in the miRNA regulatory network of Nrf2

**Fig. 7:**
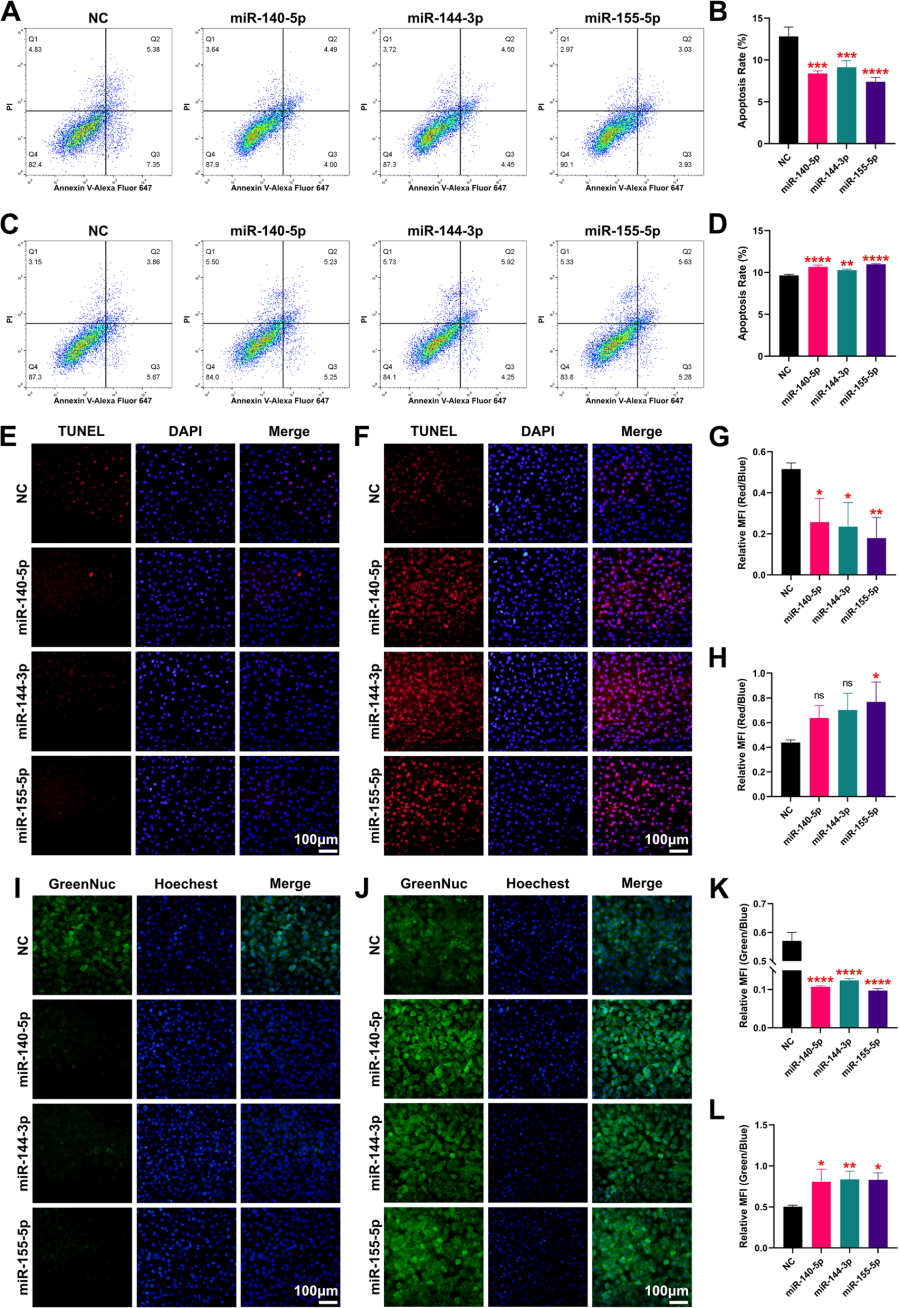
MiR-155-5p plays a key role in the miRNA regulatory network of Nrf2. **A** Apoptosis of H9C2 cells after 3 miRNA interference was detected by flow cytometry. **B** Quantitative analysis of apoptosis of H9C2 cells after 3 miRNA interference detected by flow cytometry. **C** Apoptosis of H9C2 cells after miRNA overexpression was detected by flow cytometry. **D** Quantitative analysis of apoptosis in H9C2 cells after overexpression of the 3 miRNAs was detected by flow cytometry. **E** Fluorescence micrographs of the TUNEL apoptosis assay of H9C2 cells after 3 miRNA interference. **F** Fluorescence micrographs of the TUNEL apoptosis assay of H9C2 cells after overexpression of the 3 miRNAs. **G** Quantitative analysis of the TUNEL apoptosis assay of H9C2 cells after 3 miRNA interference. **H** Quantitative analysis of the TUNEL apoptosis assay of H9C2 cells after overexpression of the 3 miRNAs. **I** Fluorescence micrographs of the caspase 3 activity assay of H9C2 cells after 3 miRNA interference. **J** Fluorescence micrographs of the caspase 3 activity assay of H9C2 cells after overexpression of the 3 miRNAs. **K** Quantitative analysis of the caspase 3 activity assay of H9C2 cells after 3 miRNA interference. **L** Quantitative analysis of the caspase 3 activity assay of H9C2 cells after overexpression of the 3 miRNAs. *ns* no significance; *P < 0.05; **P < 0.01; *** P < 0.001; **** P < 0.0001

To further confirm these findings, we examined the apoptosis, TUNEL apoptosis and caspase 3 activity of MIRI-mimicked H9C2 cells by flow cytometry after interference/overexpression of these three miRNAs. The results showed that H9C2 cell apoptosis was significantly reduced after disruption of these three miRNAs (Fig. 7A–B), and conversely, H9C2 cell apoptosis was significantly increased after overexpression of these three miRNAs (Fig. 7C–D). Meanwhile, TUNEL apoptosis of H9C2 cells was significantly decreased after interference with these three miRNAs, and conversely, it was significantly increased after overexpression of miR-155-5p (Fig. 7E–H). In addition, caspase 3 activity in H9C2 cells was significantly decreased after interference with these three miRNAs, and conversely, caspase 3 activity in H9C2 cells was significantly increased after overexpression of these three miRNAs (Fig. 7I–L). It is also noteworthy that among these three miRNAs, interference with miR-155-5p showed the best therapeutic effect.

The above results suggest that miR-155-5p would be the best choice if PMVs@PLGA complexes are to be used as vectors to carry an inhibitor of a certain miRNA to interfere with this miRNA in vivo, thus indirectly enhancing the expression of Nrf2 and ultimately playing a therapeutic role in the MIRI process.

### Use of PMVs@PLGA complexes as vectors for therapeutic roles in the MIRI process

**Fig. 8:**
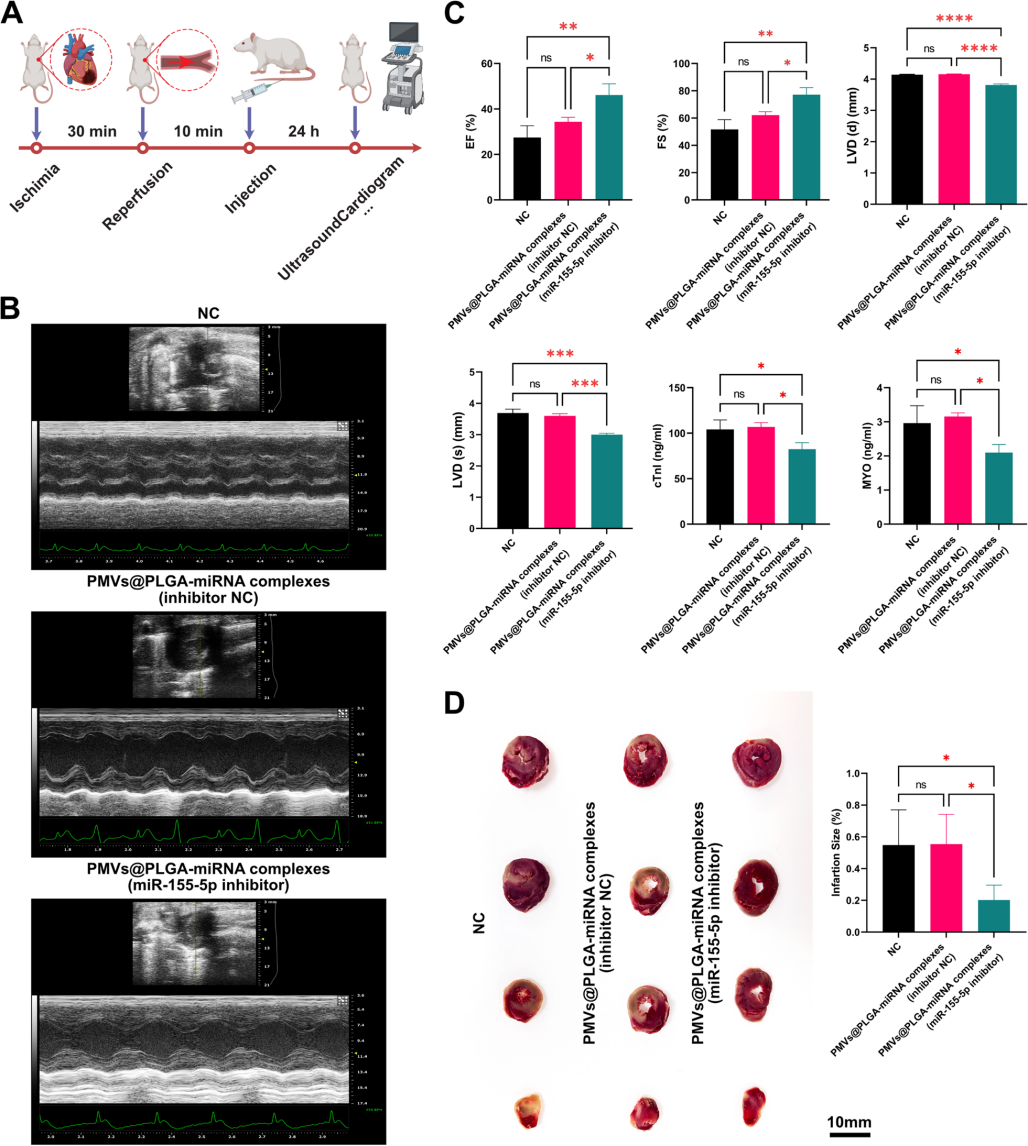
Use of PMVs@PLGA complexes as vectors for therapeutic roles in the MIRI process (1). **A** Schematic diagram to validate the efficacy of PMVs@PLGA complexes as vectors for the treatment of MIRI. **B** Images of echocardiograms after treatment of MIRI with PMVs@PLGA complexes carrying the inhibitor of miR-155-5p (antagomir). **C** Quantitative analysis of echocardiographic findings and detection of biomarkers of myocardial injury. **D** Images of TTC staining of myocardial tissue and quantitative analysis (scale bars indicate 10 mm). *ns* no significance; *P < 0.05; **P < 0.01; *** P < 0.001; **** P < 0.0001

The previous experimental results have convincingly demonstrated that PMVs@PLGA complexes are safe, nontoxic and have good transfection efficiency as a novel vector and that miR-155-5p would be the best choice as a target in vivo for the treatment of MIRI. Therefore, in in vivo experiments, we constructed an antagomir of miR-155-5p and assembled it into PMVs@PLGA-miRNA complexes (miR-155-5p inhibitor) for the treatment of MIRI, while using an equal volume of PBS as NC and again assembling the inhibitor NC into PMVs@PLGA complexes, forming PMVs@PLGA-miRNA complexes (inhibitor NC), which were injected into the rats after 10 min of reperfusion as a second control, and the treatment effect was observed after 24 h of reperfusion (Fig. 8A).

The echocardiogram results showed a significant improvement in cardiac function in rats after targeted miR-155-5p treatment (Fig. 8B). By analysing various outcome data from the echocardiogram, we obtained the following results: relative to the two control groups, treatment with PMVs@PLGA-miRNA complexes (miR-155-5p inhibitor) resulted in a significant recovery of ejection fraction (EF) and fractional shortening (FS) was significantly increased, while diastolic left ventricular diameter (LVD(d)) and systolic left ventricular diameter (LVD(s)) were significantly decreased, along with cTnI and MYO, markers of myocardial injury (Fig. 8C). This suggests that treatment with PMVs@PLGA-miRNA complexes (miR-155-5p inhibitor) resulted in improved cardiac function and reduced myocardial injury after MIRI. We removed the hearts of the rats after death for TTC staining and again found that treatment with PMVs@PLGA-miRNA complexes (miR-155-5p inhibitor) resulted in reduced infarct size and a lesser degree of cardiac injury (Fig. 8D).

**Fig. 9:**
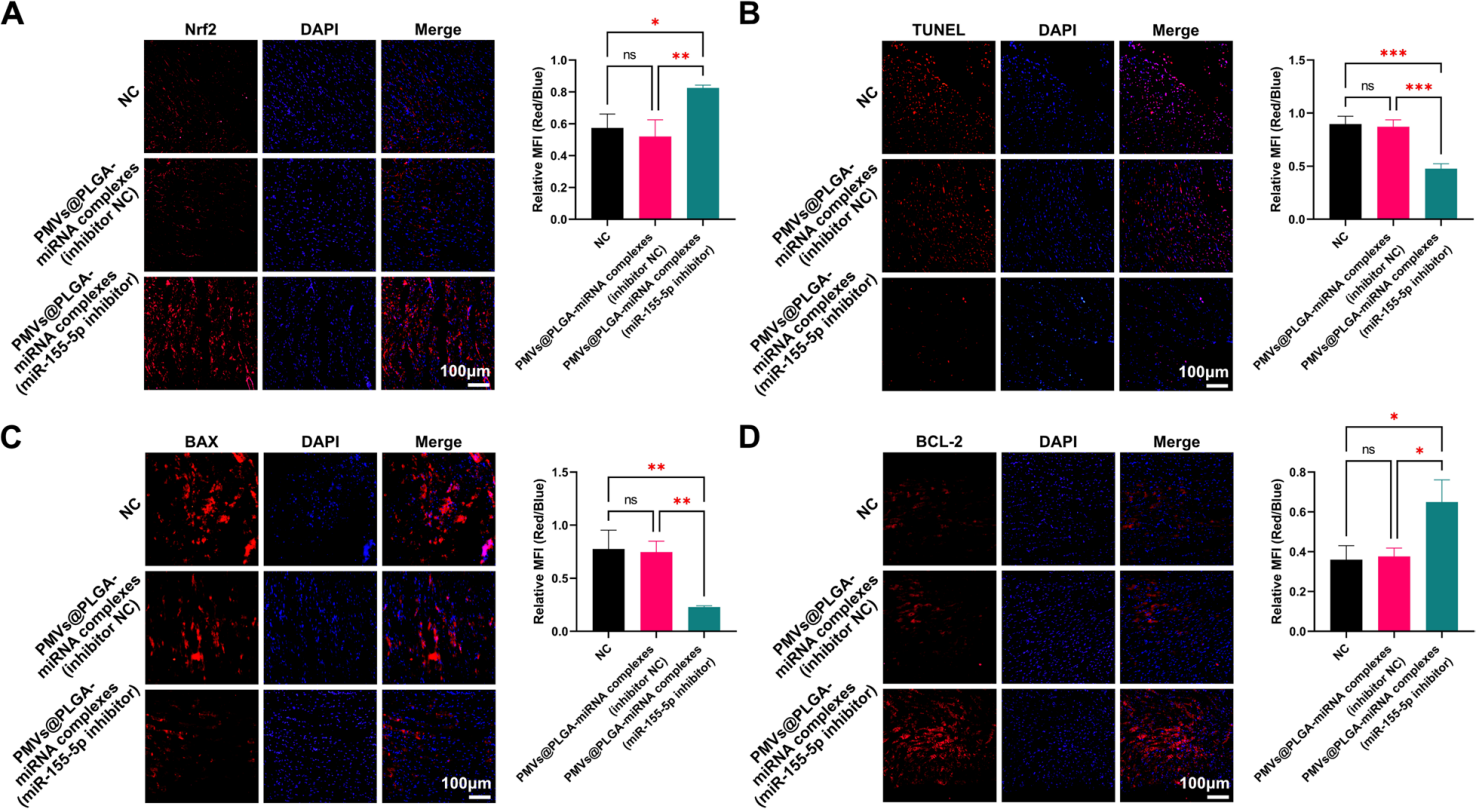
Use of PMVs@PLGA complexes as vectors for therapeutic roles in the MIRI process (2). **A** IF assay and quantitative analysis of Nrf2 expression in myocardial tissues. **B** Detection and quantification of TUNEL apoptosis in myocardial tissue. **C** IF assay and quantification of BAX expression in myocardial tissues. **D** IF assay and quantification of BCL-2 expression in myocardial tissues (scale bars indicate 100 μm). *ns* no significance; *P < 0.05; **P < 0.01; *** P < 0.001

Subsequently, we carried out a further series of tests at the molecular level. The IF results showed a significant rebound in myocardial Nrf2 expression after treatment with PMVs@PLGA-miRNA complexes (miR-155-5p inhibitor), which strongly suggests the effectiveness of PMVs@PLGA complexes as a therapeutic vector and that the use of miR-155-5p as a therapeutic target is a good strategy (Fig. 9A). We also examined TUNEL apoptosis in cardiomyocytes and found a reduction in cardiomyocyte TUNEL apoptosis following treatment with PMVs@PLGA-miRNA complexes (miR-155-5p inhibitor) (Fig. 9B). Furthermore, by IF detection of BAX and BCL-2, we found that BAX expression decreased and BCL-2 expression increased in cardiomyocytes after treatment with PMVs@PLGA-miRNA complexes (miR-155-5p inhibitor) (Fig. 9C–D). All of these results suggest that in vivo treatment with PMVs@PLGA complexes as vectors targeting miR-155-5p can effectively upregulate Nrf2 expression and significantly prevent cardiomyocyte apoptosis during MIRI. In summary, PMVs@PLGA-miRNA complexes (miR-155-5p inhibitor) are a potential therapeutic modality for MIRI.

## Discussion

Nrf2 has traditionally been regarded as a key regulator for maintaining redox balance and is involved in initiating the transcriptional expression of downstream antioxidant enzymes [11]. In addition, Nrf2 can be regulated in various ways. Nrf2/Keap1/ARE is an important signalling pathway that reduces the area of myocardial infarction and protects heart function after MIRI [35]. We have also confirmed in previous studies that the activation of Nrf2 can reduce oxidative stress during MIRI by inducing the secretion of anti-inflammatory factors [34]. A large number of studies have shown that ischaemic preconditioning and ischaemic postprocessing have obvious protective effects against MIRI, and the potential role of the Nrf2 signalling pathway in cardioprotection is worth exploring. However, how to effectively intervene in the expression of Nrf2 in the body has always been a challenge in MIRI-related research. This study is a continuation of our previous research work. Using miRNAs that regulate Nrf2 expression as an entry point, we sought to find a key miRNA as a target and prepare a novel transfection vector for in vivo transfection to indirectly increase the expression of Nrf2 to achieve therapeutic effects.

Antagomirs, as modified small RNAs, have anti-degradation properties and a long half-life in the body and have been extensively studied for in vivo transfection [21–23]. However, it is worth noting that if an antagomir enters the body directly via intravenous injection, it will still face a variety of enzymes in the circulatory system, and it will be rapidly degraded. Second, its transfection efficiency is low, and it does not have the characteristics of slow release at all, so it cannot be used as a long-term effective treatment. Therefore, it is particularly important to study new types of vectors to carry antagomirs for in vivo transfection.

There have been many studies on the use of viruses as vectors. However, the biological safety and compatibility of the virus itself are worrisome [24, 25]. As a degradable functional polymer organic compound, PLGA has good biocompatibility, nontoxicity, good encapsulation and film-forming properties and is widely used in pharmaceuticals, medical engineering materials and modern industrial fields. At present, many studies have applied PLGA to carry various plasmids or RNA for treatment. In our previous study, we used PLGA to carry plasmids for effective tendon repair treatment [29–31]. In this study, we used PLGA as a vector carrying miRNA inhibitor/mimic/antagomir for in vivo and in vitro transfection experiments.

We found that PLGA are spherical and small in size, approximately 125 ± 5 nm. These carriers easily penetrate cell membranes and release miRNA intracellularly. The complexes also have a slow release function, which prolongs the release and duration of action of the miRNA. Transfection efficiency is an important indicator of the quality of an in vivo/external nucleic acid delivery system. In our previous studies, it was demonstrated that PLGA nanoparticles have good transfection efficiency as carriers for delivering plasmids. In the present study, we found experimentally that PLGA nanoparticles and miRNA inhibitor/mimic/antagomir had the best carriage efficiency when the mass ratio was 4:1 (N:M = 4:1). Combined with previous studies and our transfection experiments in this study, we confirmed that a transfection efficiency of over 85% was achieved using PLGA nanoparticles and that transfection of miRNA inhibitor/mimic/antagomir exhibited good interference and overexpression efficiency.

Although we have proven through a series of experiments that PLGA nanoparticles as carriers for miRNA transfection have good transfection efficiency, biological safety and compatibility, there is no doubt that PLGA nanoparticles have almost no targeting ability when transfected in vivo. This reduces its therapeutic effect. How to make PLGA nanoparticles targetable for MIRI treatment is the focus of this research.

There is no doubt that platelets will accumulate at the site of myocardial ischaemia injury during MIRI. This physiological property can be used to add targeting to PLGA nanoparticles. At present, many studies have shown that the use of cell membranes to camouflage nanoparticles can allow them to target specific tasks in the body [36–41]. In the present study, we used a suitable method to obtain platelet membranes, PMVs. By TEM observation, we found that PMVs remained relatively intact as vesicle-like structures and PLGAs as regular homogeneous spherical structures, and by assembling them together and wrapping PLGA with PMVs, we obtained a novel transfection vector: PMVs@PLGA complexes. NTA analysis showed that they had a uniform particle size, and we found it interesting that the particle size distribution of PMVs@PLGA complexes was more uniform than that of PMVs, probably because the vesicle-like structure of PMVs became stronger and more homogeneous with the support of PLGA. In addition, we found that PMVs@PLGA complexes retained almost all of the platelet-derived proteins on PMVs, including important markers on platelets, which made it possible for the PMVs@PLGA complexes to retain the biological targeting of platelets themselves in vivo.

We verified the combined state of PMVs and PLGA once again after labelling them with fluorescent groups and verified the particle uptake efficiency of all three, PMVs, PLGA and PMVs@PLGA complexes, by flow cytometry, and found that the particle uptake efficiency of PMVs, PLGA, and PMVs@PLGA complexes, after combination, reached over 85%. The particle uptake efficiency was not affected by the structure.

Furthermore, we used a series of experiments, such as CCK-8 assays, flow cytometry assays for apoptosis, TUNEL apoptosis assays, caspase 3 activity assays, ROS, total SOD activity, MDA, GPx enzyme activity and LDH cytotoxicity, to demonstrate that both PMVs, PLGA, and the assembled PMVs@PLGA complexes were safe and nontoxic. After further verification that it did not cause haemolysis, we characterized the PMVs@PLGA complexes in vivo and found that they had a fast hepatic and renal metabolism rate; low cardiac, hepatic and renal toxicity; and good targeting to sites of myocardial injury. The PMVs@PLGA complexes were found to be highly enriched at the site of myocardial injury during MIRI and are a potentially excellent vehicle for MIRI therapy.

After a series of experiments, we finally identified the best target for this study: miR-155-5p. When we transfected miR-155-5p with PMVs@PLGA complexes in vitro, we effectively increased the expression of Nrf2, and MIRI-mimicked H9C2 cells showed a significant decrease in apoptosis, a significant decrease in ROS, MDA and LDH cytotoxicity, and a significant increase in total SOD activity and GPx enzyme activity. Thus, we constructed an antagomir of miR-155-5p and assembled it into PMVs@PLGA complexes, and after treatment, the echocardiographic indices of rats, biomarkers of myocardial injury (cTnI, MYO) and TTC staining showed improvements. Furthermore, at the molecular level, the expression of Nrf2 was increased, TUNEL apoptosis and BAX expression were decreased, and BCL-2 expression was increased. All of the above findings suggest that our use of PMVs@PLGA complexes harbouring inhibitors of miR-155-5p has considerable therapeutic efficacy in vivo. There have been some other studies that apply a fusion membrane to camouflage nanoparticles, which have achieved better targeting and biocompatibility. In the future, we also plan to fuse myocardial cell membranes and platelet membranes to disguise nanoparticles for MIRI treatment.

## Conclusions

In this study, we used a novel approach to deliver miRNA inhibitors in vivo by platelet membranes camouflaged with PLGA nanoparticles (i.e., PMVs@PLGA complexes). We found that in vitro, the PMVs@PLGA complexes acted as a novel transfection vector to efficiently transfect miRNA inhibitors into cells, and they played an important protective role in the process of MIRI. More importantly, in vivo, the PMVs@PLGA complexes well preserved the targeting of platelets, allowing them to target the myocardium and reduce apoptosis after the onset of MIRI. Our findings suggest a promising biotherapeutic approach that could be applied to the treatment of MIRI.

## Supplementary Information

**Additional file 1: Figure S1.** Toxicity assessment of PMVs@PLGA complexes on cardiac muscle tissue. **A** TUNEL apoptosis assay of cardiomyocytes after targeting the PMVs@PLGA complexes to the heart of SD MIRI model rats. **B** BAX IF assay of cardiomyocytes after targeting the PMVs@PLGA complexes to the heart of SD MIRI model rats. **C** BCL-2 IF in cardiomyocytes after targeting the PMVs@PLGA complexes to the heart of SD MIRI model rats (scale bars indicate 100 μm).

**Additional file 2: Figure S2.** Schematic representation of the sequences of the dual luciferase reporter gene vectors for the 4 miRNAs.

## Data Availability

Not applicable.
