# Correction: Platelet membrane-camouflaged nanoparticles carry microRNA inhibitor against myocardial ischaemia‒reperfusion injury

**DOI:** 10.1186/s12951-026-04571-3

**Published:** 2026-05-28

**Authors:** Tianyi Wang, Tingting Zhou, Mingming Xu, Shuo Wang, Anqi Wu, Mingyang Zhang, You Lang Zhou, Jiahai Shi

**Affiliations:** 1 Nantong Key Laboratory of Translational Medicine in Cardiothoracic Diseases, and Research Institution of Translational Medicine in Cardiothoracic Diseases, Affiliated Hospital of Nantong University, NO.20, Xisi Road, Nantong, 226001 Jiangsu China; 2 Department of Thoracic Surgery, Affiliated Hospital of Nantong University, NO.20, Xisi Road, Nantong, 226001 Jiangsu China; 3 Department of Forensic Sciences, Soochow University, NO.178, Ganjiang Road, Suzhou, 215000 Jiangsu China; 4 Research Center of Clinical Medicine, Affiliated Hospital of Nantong University, NO.20, Xisi Road, Nantong, 226001 Jiangsu China; 5 School of Public Health, Nantong University, NO.9, Seyuan Road, Nantong, 226019 Jiangsu China

**Correction: Journal of Nanobiotechnology (2022) 20:434**

10.1186/s12951-022-01639-8

The author has noticed some errors in Fig. 7E resulting from the incorrect use of images (due to incorrect grouping of images during photography) and have made the necessary corrections. Specifically, the authors have corrected the corresponding image for miR-140-5p in Fig. 7E. All in vivo experiments were performed using 3 rats per group. For in vitro experiments, the assays were performed in 3 biological replicates, except Figs. 3B-E and 7E-L, where only technical replicates were performed. We apologize for the inconvenience caused to readers by the mistakes.

Incorrect Fig. 7.


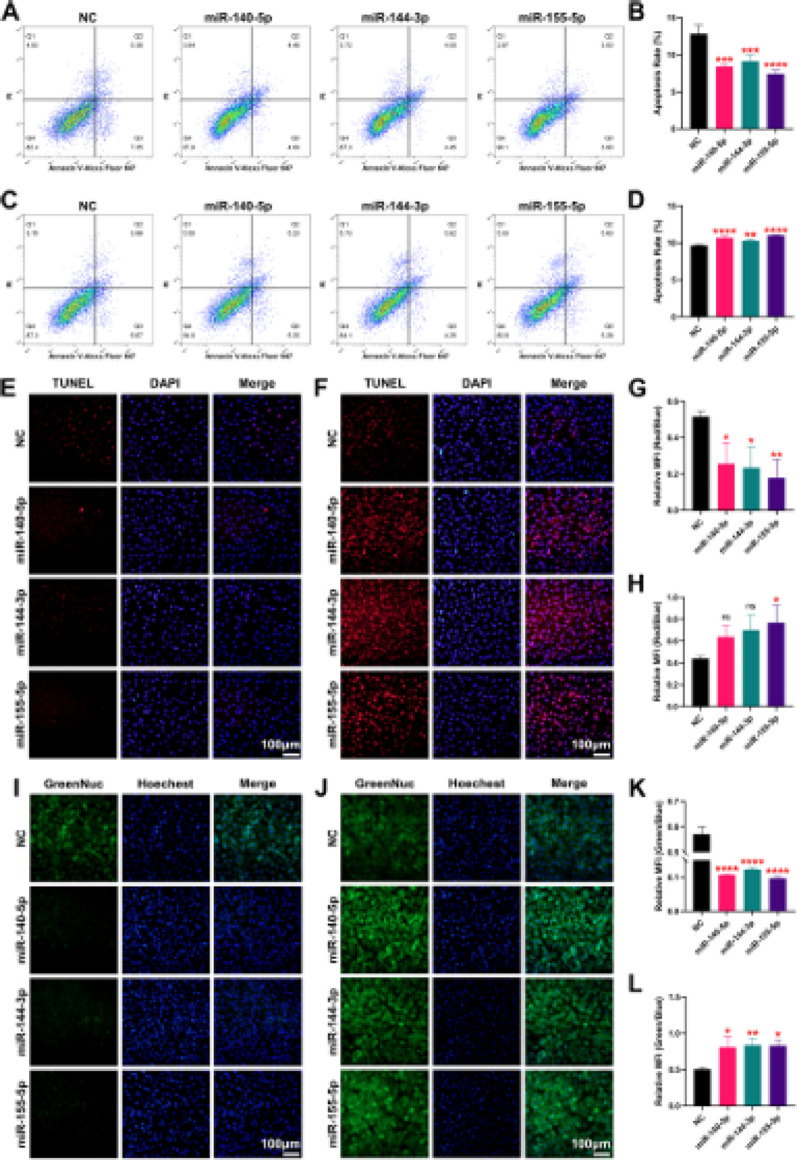


Correct Fig. 7.

**Fig. 7:**
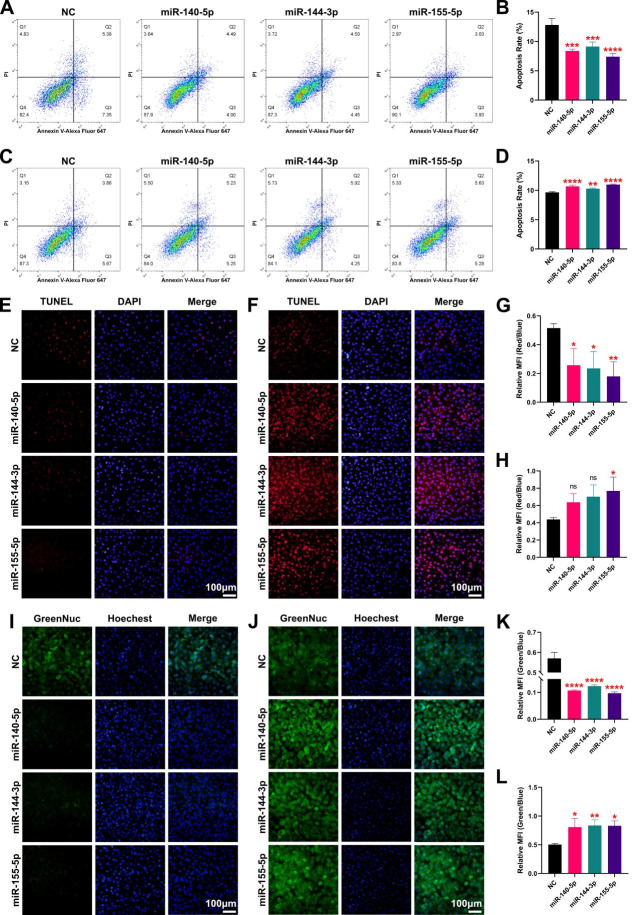
MiR-155-5p plays a key role in the miRNA regulatory network of Nrf2. **A** Apoptosis of H9C2 cells after 3 miRNA interference was detected by flow cytometry. **B** Quantitative analysis of apoptosis of H9C2 cells after 3 miRNA interference detected by flow cytometry. **C** Apoptosis of H9C2 cells after miRNA overexpression was detected by flow cytometry. **D** Quantitative analysis of apoptosis in H9C2 cells after overexpression of the 3 miRNAs was detected by flow cytometry. **E** Fluorescence micrographs of the TUNEL apoptosis assay of H9C2 cells after 3 miRNA interference. **F** Fluorescence micrographs of the TUNEL apoptosis assay of H9C2 cells after overexpression of the 3 miRNAs. **G** Quantitative analysis of the TUNEL apoptosis assay of H9C2 cells after 3 miRNA interference. **H** Quantitative analysis of the TUNEL apoptosis assay of H9C2 cells after overexpression of the 3 miRNAs. **I** Fluorescence micrographs of the caspase 3 activity assay of H9C2 cells after 3 miRNA interference. **J** Fluorescence micrographs of the caspase 3 activity assay of H9C2 cells after overexpression of the 3 miRNAs. **K** Quantitative analysis of the caspase 3 activity assay of H9C2 cells after 3 miRNA interference. **L** Quantitative analysis of the caspase 3 activity assay of H9C2 cells after overexpression of the 3 miRNAs. ns no significance; *P < 0.05; **P < 0.01; *** *P* < 0.001; **** *P* < 0.0001

The original article has been corrected.

