# Supplementary figures and images for: Platelet membrane-camouflaged nanoparticles carry microRNA inhibitor against myocardial ischaemia‒reperfusion injury
Source: J Nanobiotechnology. 2022 Oct 4;20:434. doi: 10.1186/s12951-022-01639-8 (PMC9531416; doi:10.1186/s12951-022-01639-8)

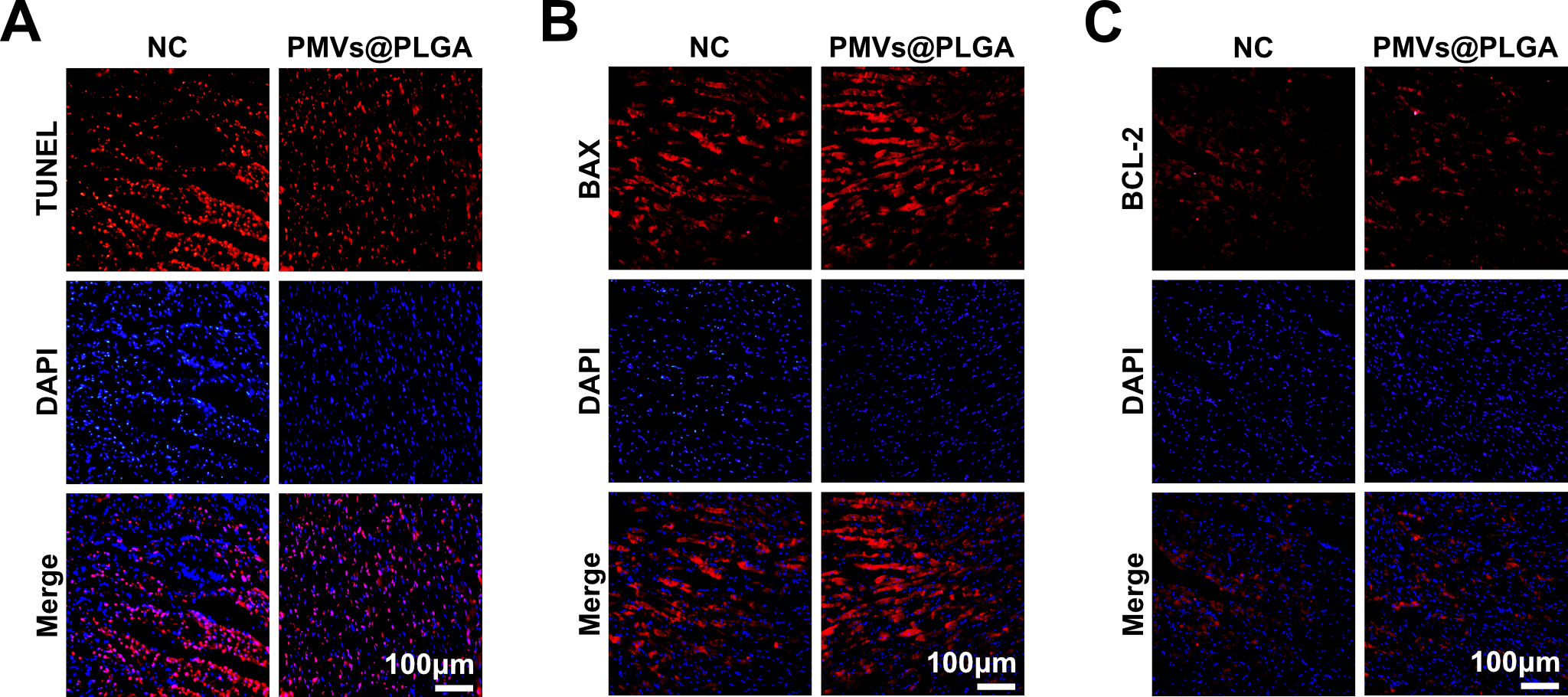

Supplement: Supplementary file 1 — Additional file 1: Figure S1. Toxicity assessment of PMVs@PLGA complexes on cardiac muscle tissue. A TUNEL apoptosis assay of cardiomyocytes after targeting the PMVs@PLGA complexes to the heart of SD MIRI model rats. B BAX IF assay of cardiomyocytes after targeting the PMVs@PLGA complexes to the heart of SD MIRI model rats. C BCL-2 IF in cardiomyocytes after targeting the PMVs@PLGA complexes to the heart of SD MIRI model rats (scale bars indicate 100 μm). [file 12951_2022_1639_MOESM1_ESM.tif]

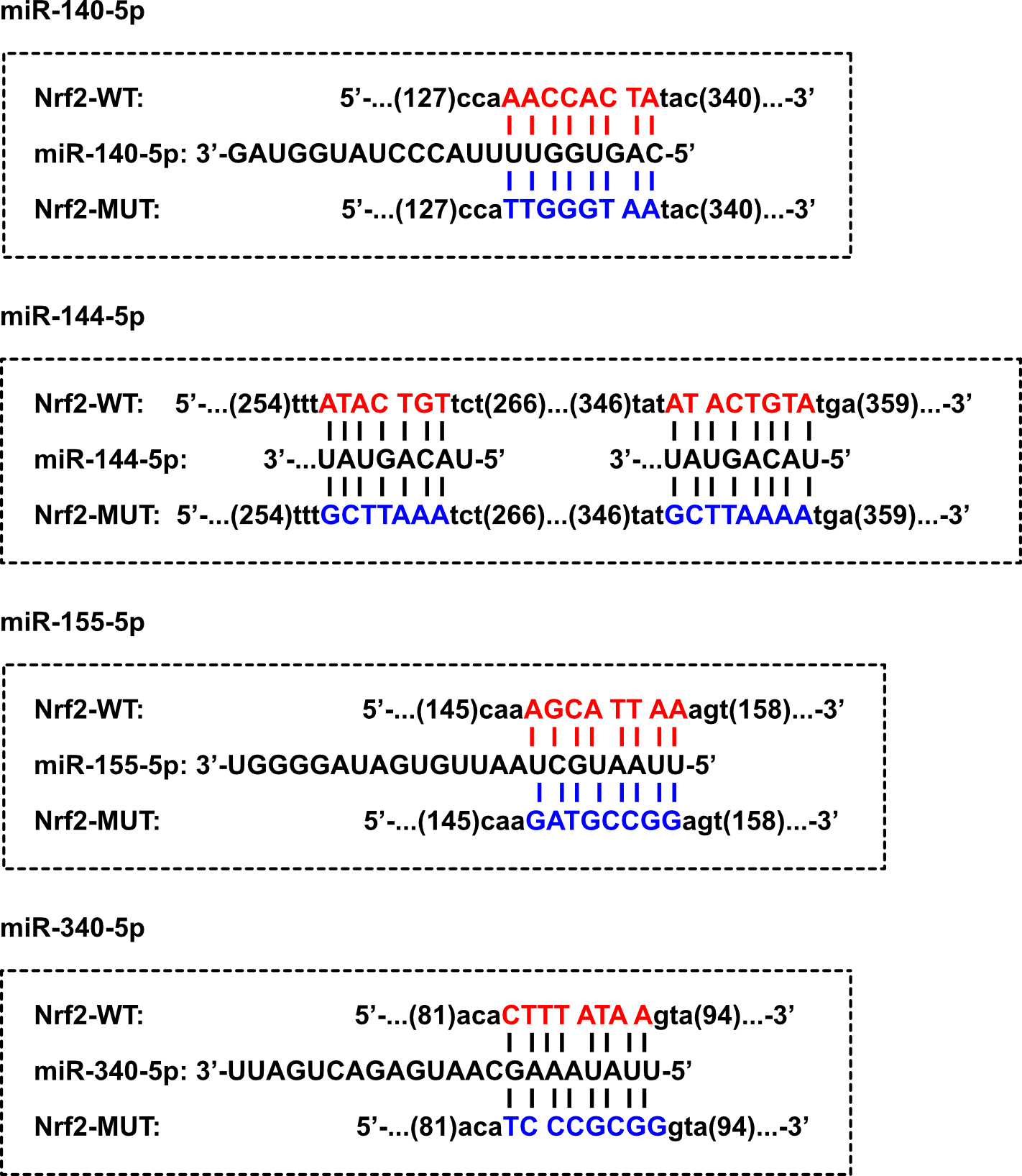

Supplement: Supplementary file 2 — Additional file 2: Figure S2. Schematic representation of the sequences of the dual luciferase reporter gene vectors for the 4 miRNAs. [file 12951_2022_1639_MOESM2_ESM.tif]
